# Three distinct regions of cRaf kinase domain interact with membrane

**DOI:** 10.1038/s41598-019-38770-w

**Published:** 2019-02-14

**Authors:** Priyanka Prakash, John F. Hancock, Alemayehu A. Gorfe

**Affiliations:** Department of Integrative Biology and Pharmacology, McGovern Medical School, University of Texas Health Science Center at Houston, 6431 Fannin St., Houston, Texas 77030 USA

## Abstract

Raf kinases are downstream effectors of small GTPase Ras. Mutations in Ras and Raf are associated with a variety of cancers and genetic disorders. Of the three Raf isoforms, cRaf is most frequently involved in tumor initiation by Ras. Cytosolic Raf is auto-inhibited and becomes active upon recruitment to the plasma membrane. Since the catalytic domain of Raf is its kinase domain, we ask the following: does the kinase domain of Raf has potential to interact with membrane and if yes, what role does the membrane interaction play? We present a model of cRaf kinase domain in complex with a heterogeneous membrane bilayer using atomistic molecular dynamics simulation. We show that the kinase domain of cRaf has three distinct membrane-interacting regions: a polybasic motif (R.RKTR) from the regulatory αC-helix, an aromatic/hydrophobic cluster from the N-terminal acidic region (NtA) and positively charged/aromatic cluster from the activation segment (AS). We show that residues from these regions form an extended membrane-interacting surface that resembles the membrane-interacting residues from known membrane-binding domains. Activating phosphorylatable regions (NtA and AS), make direct contact with the membrane whereas R.RKTR forms specific multivalent salt bridges with PA. PA lipids dwell for longer times around the R.RKTR motif. Our results suggest that membrane interaction of monomeric cRaf kinase domain likely orchestrates the Raf activation process and modulates its function. We show that R.RKTR is a hotspot that interacts with membrane when cRaf is monomeric and becomes part of the interface upon Raf dimerization. We propose that in terms of utilizing a specific hotspot to form membrane interaction and dimer formation, both Raf and its upstream binding partner KRas, are similar.

## Introduction

**Figure 1:**
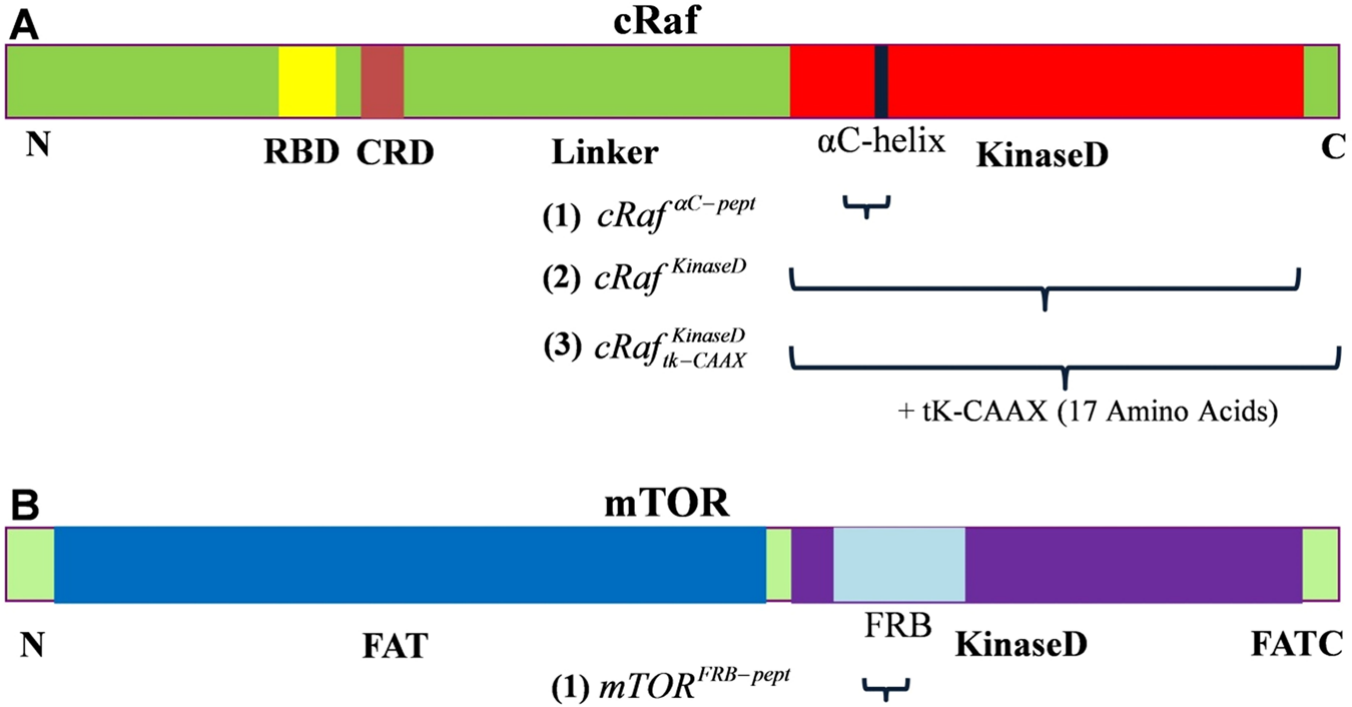
The domain architecture of cRaf **(A)** and mTOR **(B)** kinases. Raf has a Ras-binding domain (RBD), a cysteine-rich domain (CRD) and a catalytic kinase domain (KinaseD) containing an αC-helix in the N-lobe of the KinaseD. The N-terminus of mTOR is made up of FAT domain followed by a kinase domain, N-lobe of which has a FRB (FKBP12-Rapamycin-binding) domain. The membrane-bound peptides and protein domains which are subjected to MD simulations are shown by numbers (1), (2) and (3).

Raf kinases are downstream binding partners of Ras^1^. Ras-Raf activates MAPK signaling pathway involved in a variety of cell signaling processes^2^. Ras shuttles between GTP-bound or active and GDP-bound or inactive states, is prenylated, and binds with membrane for its normal function^3–5^. Mammalian Raf kinases exist in three isoforms, a-, b- and cRaf^1^. cRaf is the most important isoform involved in the tumor initiation by Ras^6,7^. GTP-bound Ras recruits Raf to the plasma membrane^8,9^. Raf is a multi-domain protein made up of a Ras-binding domain (RBD), a cysteine-rich domain (CRD), a linker region, and a catalytic kinase domain (KinaseD) (Fig. 1). The functional unit of Raf is a dimer formed by the kinase domain of two Raf protomers^10^. Either a homo- or hetero-dimer is formed by almost all possible combinations of the three isoforms with heterodimers specifically between cRaf and bRaf being the most common^11,12^. The N-lobe of Raf KinaseD consists of a regulatory helix, αC-helix, and functional modulation of Raf kinase activity by the spatial location of αC-helix is well-known^13,14^. αC-helix in cRaf:KinaseD harbors an RKTR motif that is directly involved in the Raf dimer interface formation^10^. RBD and CRD together form an N-terminal regulatory region^15^. Cytosolic Raf is inactive, and exists in an auto-inhibited state wherein the N-terminal regulatory region engages the C-terminal kinase domain, thereby masking and rendering it inactive^15^. The auto-inhibition is released upon a Ras-dependent recruitment of Raf to the plasma membrane evoked by interaction of Raf-RBD with Ras-GTP, leading to unmasking of the kinase domain^8,9^.

Studies show that tethering cRaf with the minimal membrane anchor of K-Ras containing prenylation motif, CAAX, henceforth referred to as tK-CAAX, results in the recruitment of Raf to the plasma membrane in a Ras-independent manner^8,9^. Tethering tK-CAAX to an isolated kinase domain of Raf alleviates the need for release of auto-inhibition, possibly because the N-terminal regulatory region is no longer present, and this construct mimics a Ras-induced state of Raf^16^. The crystal structure of RBD^17^, CRD^18^ and kinaseD^11^ of cRaf have been solved in isolation; however, the long linker region (~200 residues long) connecting the N-terminal regulatory region with the kinaseD is not yet known. While RBD’s primary role is to interact with membrane-bound GTP-Ras^19^, the CRD, which is structurally and functionally similar to a membrane-interacting C1 domain, also interacts with lipids^18,20^. Recently, complexes of Ras and Ras:Raf-RBD-CRD with membrane have been investigated using a number of methods including computational, spectroscopic and biochemical approaches^21–28^. This raises an intriguing question: does the kinase domain of Raf also interact with the membrane?

Previous studies suggested the presence of a PA (phosphatidic acid) interacting region in the cRaf:kinaseD^29,30^. A lipid-interacting region has also been observed in another kinase, mTOR (mammalian target of Rapamycin)^31^. mTOR is a downstream effector of the Ras homologue in brain (Rheb) and, like Raf, it has a catalytic kinase domain (Fig. 1)^32^. The αC helix in cRaf and the FRB domain in mTOR, which lie in the N-lobe of kinase domain, harbors this potential PA-interacting region (Fig. 1). While the spatial location of αC-helix is a well-known functional modulator of Raf kinase activity^13,14^, mutation in the PA-interacting region within the FRB domain correlated with a ~60% reduction in the mTOR downstream signaling activity^31^. This is suggestive of an important role of membrane in modulating the function of cRaf and mTOR.

Based upon these observations, we hypothesize that the kinase domain of cRaf as well as mTOR harbors a membrane-interacting region that is distinct from their previously characterized membrane interacting regions/motifs. To test this hypothesis, we first performed molecular dynamics (MD) simulations of the putative PA-interacting regions of cRaf and mTOR, and the full-length kinase domain of cRaf attached to tK-CAAX, in a binary lipid bilayer containing 12% PA and 88% PC. The latter construct ensures membrane localization in a Ras-independent manner^8,9,16^. Our results show that a polybasic R.RKTR motif from the regulatory αC-helix, an aromatic/hydrophobic cluster from the N-terminal acidic region (NtA), and aromatic/positively charged residues from the activation segment (AS) in the kinase domain of cRaf make direct contact with the membrane. R391 and 398-RKTR (R.RKTR) is PA-specific, and PA lipids dwell for long near this cluster. Polybasic cluster-PA interactions are stabilized by multivalent salt bridges. αC-helix and activating phosphorylatable regions (NtA and AS)^1^ are in direct contact with the membrane throughout the simulations. Together, these three regions project an extended membrane interacting surface containing positively charged, aromatic, and hydrophobic residues, that resembles the membrane-interacting residues from other established membrane-binding domains (e.g.^33^). The functional implication of KinaseD-membrane interaction likely lies in priming Raf for activation by properly positioning phosphorylatable regions and αC-helix for phosphorylation and dimerization, considering that membrane targeting enhances phosphorylation and that phosphorylation precedes dimerization^34,35^. To our knowledge, this is the first study that presents a detailed atomistic model of the complex between cRaf kinase domain and a heterogeneous membrane.

## Methods

We constructed a membrane bilayer patch containing 366 POPC (88%) and 50 POPA (12%) lipids using CHARMM-GUI^36^ and subjected it to equilibration as described below. Using this bilayer, molecular dynamics (MD) simulations were carried out on (1) the potential PA-interacting regions of cRaf and mTOR kinase domains (*cRaf* ^αC-*Pept*^ and *mTOR*^*FRB*-*pept*^), (2) full-length cRaf kinase domain (*cRaf* ^*KinaseD*^), and (3) full-length *cRaf* ^*KinaseD*^ glued to a modeled C-terminus of cRaf that in turn is linked to the prenylated C-terminus of K-Ras ($cRa{f}_{tk-CAAX}^{KinaseD}$) (Table S1).

### Preparation of initial models

*cRaf* ^αC-*Pept*^ *and mTOR*^*FRB*-*pept*^: The potential PA-interacting regions of cRaf (residues 389–426) and mTOR (residues 2101–2137) were taken from 3OMV^37^ and 4JSV^32^, respectively (Fig. 1). Each peptide was simulated in a PC/PA bilayer. *cRaf* ^αC-*Pept*^ simulation contained four cRaf peptides (two each in the top and bottom leaflets) and *mTOR*^*FRB*-*pept*^ simulation contained two mTOR peptides (one each in the top and bottom leaflets). For cRaf, the peptides were placed with their centers of mass approximately 60 Å apart in a box of length 120 × 120 Å in the x-y dimension. In each simulation, the peptides were positioned near the interface of a PC/PA bilayer model with their hydrophilic residues in close proximity to the lipids.

*cRaf* ^*KinaseD*^: Chain A of the kinase domain of cRaf from 3OMV^37^ was used as the starting structure (residues 340–615). This and the majority of other crystal structures of kinases have missing activation segment (AS) (residues 492–503), which was modeled using Modeller 9.11^38^. While inactive Raf kinase has an ordered AS^39^, here, our primary goal is to probe the membrane interaction of an active monomer that will likely undergo dimerization. Therefore, we selected an AS model with a completely disordered conformation. The position of ATP molecule and magnesium ions was determined by a structural alignment of the cRAF kinase domain and cyclic AMP dependent protein kinase (PDB: 4DFX^40^). The starting structure for the *cRaf* ^*KinaseD*^ - membrane complex was obtained by docking the kinase domain onto one of the membrane-bound peptides in the last snapshot of the *cRaf* ^αC-*Pept*^ simulation.

$cRa{f}_{tk-CAAX}^{KinaseD}$: Studies have shown that the kinase domain of cRaf is recruited to the plasma membrane in a Ras-independent manner if a CAAX motif of K-Ras (tK-CAAX) is attached at its C-terminus^8,9,16^. Therefore, first we modeled the C-terminus of cRaf (Fig. 1) using PEPFOLD^41^ (residues 615–648). We obtained five low energy models with a full or partial helical content. We picked the first lowest energy model, which was completely helical. Also, a protein blast search of the C-terminus sequence against protein-data bank yielded a hit with an alpha-helical structure having 50% sequence similarity with the query. Additionally, other studies have shown that the C-terminus of other kinases, including mTOR (FATC, Fig. 1), adopts a helical structure upon binding to membrane-mimetic micelles^42^. A farnesylated K-Ras lipid anchor made up of 17 C-terminal amino acids (tk-CAAX) obtained from our previous study of membrane-bound K-Ras^23^ was added onto the modeled C-terminus of cRaf. The KinaseD from this construct and the last snapshot obtained from *cRaf* ^*KinaseD*^ simulation were structurally aligned, and farnesyl was pulled inside the PA/PC membrane. This system is referred as $cRa{f}_{tk-CAAX}^{KinaseD}$ (Fig. 1, Table S1).

### Molecular dynamics simulation

The systems were energy minimized for 2000 steps with lipids and proteins fixed and equilibrated for 200 ps with the lipid phosphate and heavy atoms of proteins harmonically restrained with a force constant k = 4 kcal/mol/Å^2^. The k was gradually scaled by 0.75, 0.50, 0.25 and 0 with a time step of 1 fs. Production run was performed using a time step of 2 fs using SHAKE to restrain all bonds involving hydrogen atoms^43^. PME was used for electrostatics with 12 and 14 Å cutoffs for non-bonded interactions^44^. NPT ensemble was used with constant pressure maintained at 1 bar by the Nose-Hoover Langevin piston method and temperature of 310 K controlled by Langevin thermostat. The CHARMM36 force field for proteins^45^ and lipids^46^ was used with CMAP dihedral correction for proteins^47,48^. Simulations were run with the NAMD2.11 program^49^. *cRaf* ^αC-*Pept*^ and *mTOR*^*FRB*-*pept*^ were simulated for ~1 μs. Each of *cRaf* ^*KinaseD*^ and $cRa{f}_{tk-CAAX}^{KinaseD}$ was simulated for 500 ns (Table S1).

### Analysis

Encounter between the peptide and lipid is defined as a contact when heavy atoms of proteins are within a distance, d, of 4 Å of any heavy atom of the lipid headgroups. Residence time of PA (t_res_) around those protein residues that are in contact with the membrane for >50% of simulation time is calculated as follows:

1 $${t}_{res}\equiv (\begin{array}{ll}{t}_{f}-{t}_{i}; & d\le 4{\rm{\AA }}\\ 0; & d > 4{\rm{\AA }}\end{array})$$

where, t_i_ and t_f_ are the initial and final time with Δt = 10 ps. Aggregate residence time is defined as Σt_res._

## Results

### Sequence and structural analysis predicts a membrane-interacting region in the kinase domain of cRaf

**Figure 2:**
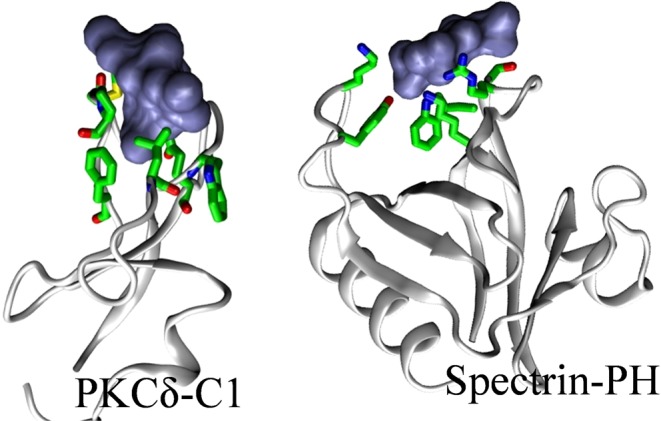
Membrane-interacting residues (green sticks) in C1 (**A**) and PH (**B**) domains of PKCδ (1PTR) and Spectrin (1BTN), respectively, in complex with phorbol acetate and inositol phosphate (blue surface). Color scheme: protein (gray), nitrogen (blue), oxygen (red), carbon (green).

There are several protein domains that have a high propensity of interacting with the membranes^33,50^. Some examples include C1, C2, PH and FYVE domains^50^. Some of these have a defined lipid-binding pocket that facilitates interaction with the membrane by recognizing specific membrane components such as PS, phosphoinositides, phorbol acetate, or even ions (e.g. PKCδ^51^, Fig. 2A)^33,50^. Others have no well-defined lipid-binding pockets but rather contain a positively charged surface patch (e.g. FERM, PDZ, PTB, ANTH, Spectrin^52^; Fig. 2B)^33,50^. Even the same domain type in different multi-domain proteins can have either a well-defined lipid binding pocket or a positively charged surface patch. For example, PH domain forms a lipid-binding pocket in SOS^53^ and a relatively solvent exposed surface patch of positively charged residues in Spectrin^52^. Note that a surface patch or pocket can be formed by the spatial proximity of residues from distal secondary structure elements (separated by double dots in Table 1).

**Table 1:** Known and predicted membrane interacting residues from membrane-binding protein domains.

Protein	Domain	Membrane-interacting residues^*^
PKCα	C2	K.K..KY..W..N
Syntaxin1A	BAR-like	KK.KY.K.RRKK
SOS	PH	K..R..K..R..FF..Y
PKCδ	C1	M.P.F.L.W.Q^#^
Kcc4p (yeast)	KA	K..K..K..K..ILFL..KK
MARK/PAR1 kinase	KA1	R.R..R..KR
Spectrin	PH	K..R.W..Y.K
cRaf	Kinase	R.RK.R^&^
mTOR	FRB-kinase	R.R.K^&^

*Single dot = residues from same secondary structure element; double dot = residues from different secondary structure elements; No dot = residues are contiguous.

^#^An exception containing hydrophobic and aromatic residues only.

^&^Predicted from our study.

We observe that, for the selected known membrane-binding domains, the membrane-interacting residues can be classified roughly into three groups: (1) largely positively charged (e.g. syntaxin 1A, MARK/PAR1), (2) positive and aromatic/hydrophobic (e.g. PKCα, SOS, spectrin) and (3) polar, aromatic and hydrophobic (e.g. PKCδ) (Table 1). In line with this, we observed a positively charged cluster, 391R-398-RKTR and 2108-RRISK, in the suggested PA-interacting region of cRaf and mTOR, respectively (Table 1). Aromatic and/or hydrophobic residues either alone or in combination with positively charged residues are not unexpected given that the aromatic residues prefer lipid-water interfaces while hydrophobic residues can insert into the membrane, as suggested recently for a newly identified membrane-interacting kinase associated domain-1 (KA1)^54,55^.

**Figure 3:**
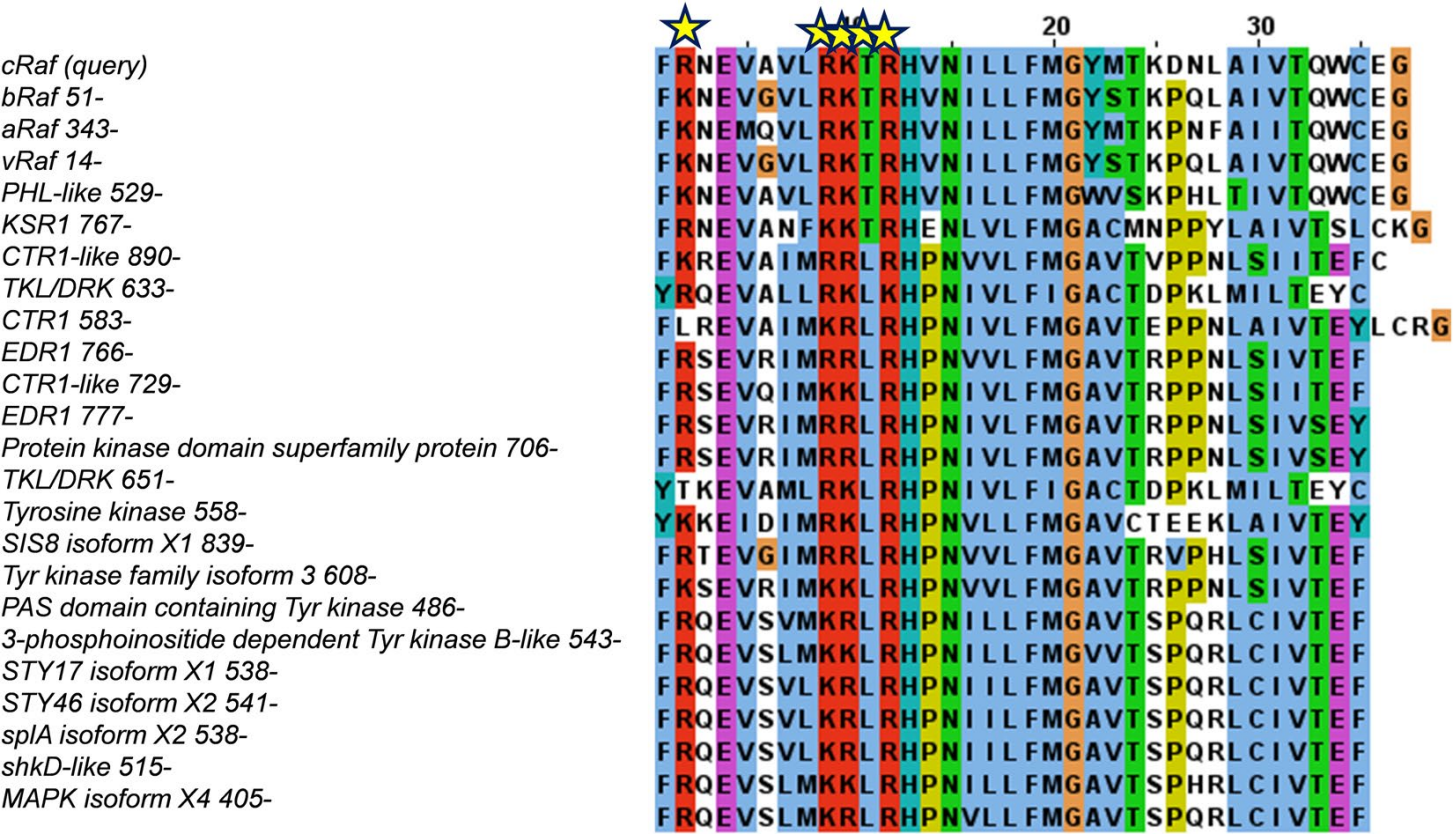
Multiple sequence alignment of selected sequences. A conserved putative membrane-interacting sequence motif, **R**xEx^4^**R**(K)**K**(R)T(L)**R**, is observed among all Raf kinases. (see Supplementary Information).

Furthermore, subjecting the PA-interacting region of cRaf (390-FRNEVAVLRKTRHVNILLFMGYMTKDNLAIVTQWCEG) to a blast search against the non-redundant protein database identified other serine/threonine kinases (STK) and STK- or Raf-like kinases with a similar region, highlighting a conserved sequence motif containing a positively charged residue cluster, **R**xEx^4^**R**(K)**K**(R)T(L)**R**, where x is any amino acid (Fig. 3). All Raf isoforms contain the motif R(K)xEx^4^RKTR (Fig. 3). The sequences analyzed here are from diverse organisms ranging from plants, drosophila to humans (Table S2). Direct interaction of the catalytic kinase domain of aRaf and cRaf with the membrane has been suggested^29,56^ and evidence exists for membrane localization of two other kinases: KSR and CTR1^57,58^. However, the kinase domains of KSR and CTR1 are not shown to interact with the membrane directly but rather via their N-terminal region either directly (like Raf-CRD) or indirectly (like Raf-RBD). Based upon this and together with the presence of a putative membrane-interacting sequence motif in most kinase domains (Fig. 3), one can speculate that direct membrane interaction of kinase domains is generalizable.

In addition to the involvement of **R**xEx^4^**R**(K)**K**(R)T(L)**R** in the formation of dimer interface in the Raf kinases^10^ and likely in other kinases as well, the combined observation from our sequence and structure-based analysis is suggestive of the presence of a putative membrane-interacting region in the catalytic domain of kinases in general, and specifically in cRaf and mTOR kinases (Table 1). Focusing on the polybasic residues of cRaf, henceforth, we write RxEx^4^RKTR as R.RKTR.

### R.RKTR in cRaf and RRISK in mTOR are membrane-interacting motifs

We performed MD simulations on the potential PA-interacting segments of cRaf and mTOR, *cRaf* ^αC-*Pept*^ and *mTOR*^*FRB*-*pept*^, respectively, in a PA/PC bilayer model membrane. We ensured that the individual peptides in *cRaf* ^αC-*Pept*^ and *mTOR*^*FRB*-*pept*^ do not interact amongst themselves and thus can be considered as four independent peptide-membrane systems (Fig. S1). Since the lipid distribution is also symmetric (each leaflet contains 183 randomly placed POPC and 25 POPA lipids), the addition of four or two peptides did not perturb the global structure of the bilayer. For example, the average area per lipid was ~62.4 Å^2^, which is in agreement with POPC-dominated binary lipid mixtures^59^. Therefore, we performed our analysis by taking an average over the four (cRaf) and the two (mTOR) peptides, unless mentioned otherwise.

**Figure 4:**
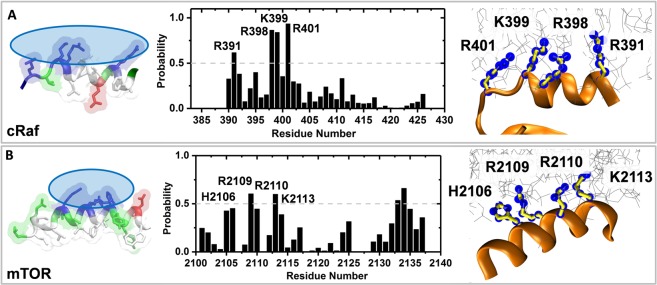
Peptide-lipid interactions in cRaf (**A**) and mTOR. **(B**) The helices in both *cRaf* ^αC-*Pept*^ and *mTOR*^*FRB*-*pept*^ are amphipathic (left). Average probability of contact between protein and lipids (middle). Contact is defined when any protein heavy atoms is within 4Å of heavy atoms of lipid headgroups. A snapshot showing residues in contact with membrane for >50% of the simulation length (right). Color scheme: (left)-charged surface (blue transparent ovals), basic residues (blue), acidic residues (red), hydrophobic (white), others (green); (right)-helical region (orange), carbon (yellow), residues in contact with membrane are highlighted by blue points. The membrane is shown in gray sticks.

Both *cRaf* ^αC-*Pept*^ and *mTOR*^*FRB*-*pept*^ form stable interactions with the membrane throughout the simulation, with 6 to 12 residues being in direct contact with the PC or PA lipids (Fig. S2). In the beginning of the simulations, the peptides were placed with their hydrophilic residues facing the membrane. The peptides were further drawn towards the membrane during the initial simulation stage. Therefore, though we did not expect an increase in the number of residues that are in contact with lipids with time, we wanted to assess how stable the peptide-membrane interaction is, and if it occurs via specific residues. To do so, we focused on the helical region of both *cRaf* ^αC-*Pept*^ and *mTOR*^*FRB*-*pept*^ that contains a polar face made up of positively charged residues that may interact with the negatively charged environment provided by the anionic lipids (Fig. 4, left). All peptides in *cRaf* ^αC-*Pept*^ and *mTOR*^*FRB*-*pept*^ remain bound to membrane throughout the simulation via this charged surface (Fig. 4, middle). One of the peptides in *mTOR*^*FRB*-*pept*^ dissociated and re-associated with the membrane (and hence the lower average probability of contact in Fig. 4, middle), but it remained stably bound for the last ~500 ns. While re-associating, it made interactions via the same charged surface as before (Fig. 4, left and middle).

Charged residues from the helical region involved in stable protein-lipid contacts are R391, R398, K399 and R401 in cRaf and H2106, R2109, R2110 and K2113 in mTOR (Fig. 4, middle). The former corresponds to our sequence motif, R.RKTR, and the latter, to the RRISK of mTOR. In the latter, R2134, a non-helical residue, also interacts with membrane but here we focus primarily on the helical region. This is because non-helical regions, owing to their higher flexibility, may show different dynamics when the full-length protein is considered. The helical residues made stable interactions with the POPA and POPC phospholipids for >50% of the simulation time (Fig. 4, middle and right). Henceforth, we refer to R391 and 398-RKTR (R.RKTR) either as a polybasic cluster or membrane-interacting sequence motif of cRaf and use these two terms interchangeably throughout the text. Similarly, R2109, R2110 and K2113 of mTOR is referred to as mTOR’s polybasic cluster (Fig. 4, middle). The interaction pattern resembles a perfect amphipathic helix where hydrophobic residues L2103, Y2104, V2107, F2108, I2111, L2115 do not interact with the membrane and are involved in hydrophobic packing interaction in the four-helix bundle of the FRB domain.

Recently, studies suggesting a role of membrane in modulating the function of membrane-bound small GTPases have emerged. This concept is termed as membrane orientation dynamics (or membrane reorientation) according to which specific protein surfaces, often containing positively charged residues, interact directly and transiently with the membrane^21,60^. As a result, the proteins adopt distinct and specific orientation with respect to the membrane (e.g.^60^). These distinct orientations in turn have been related to variable functional outputs. Membrane orientation dynamics has been observed in Ras (N-, H- and K-Ras)^21–24^, other members of the Ras superfamily such as Arf and Rheb^61,62^, and the membrane-interacting CRD domain of Raf kinases^25,28^. The protein-membrane interaction during membrane reorientation serves as a secondary interaction since the specific membrane-anchoring motifs (such as CAAX motif in K-Ras) provides the primary interaction with membrane. The helical region harboring the polybasic cluster in both *cRaf* ^αC-*Pept*^ and *mTOR*^*FRB*-*pept*^ adopts a perpendicular orientation with respect to the membrane normal (Fig. S3). The orientation of the helical region is found conserved in the full-length kinase domain, $cRa{f}_{tk-CAAX}^{KinaseD}$, and possibly modulates the activation of Raf (section 4). We believe that under physiological conditions, the observed interaction between αC-helix of cRaf and FRB domain of mTOR with the membrane is secondary since membrane-interacting motifs provide the primary attachment in both mTOR^42,63,64^ and cRaf^5,17,29,65,66^.

Our results show that the polybasic cluster in cRaf and mTOR acts as membrane-interacting motif. This strengthens our hypothesis that kinase domain has a membrane-interacting region. In subsequent sections, we focus our attention on cRaf.

### R.RKTR motif is PA-specific

**Figure 5:**
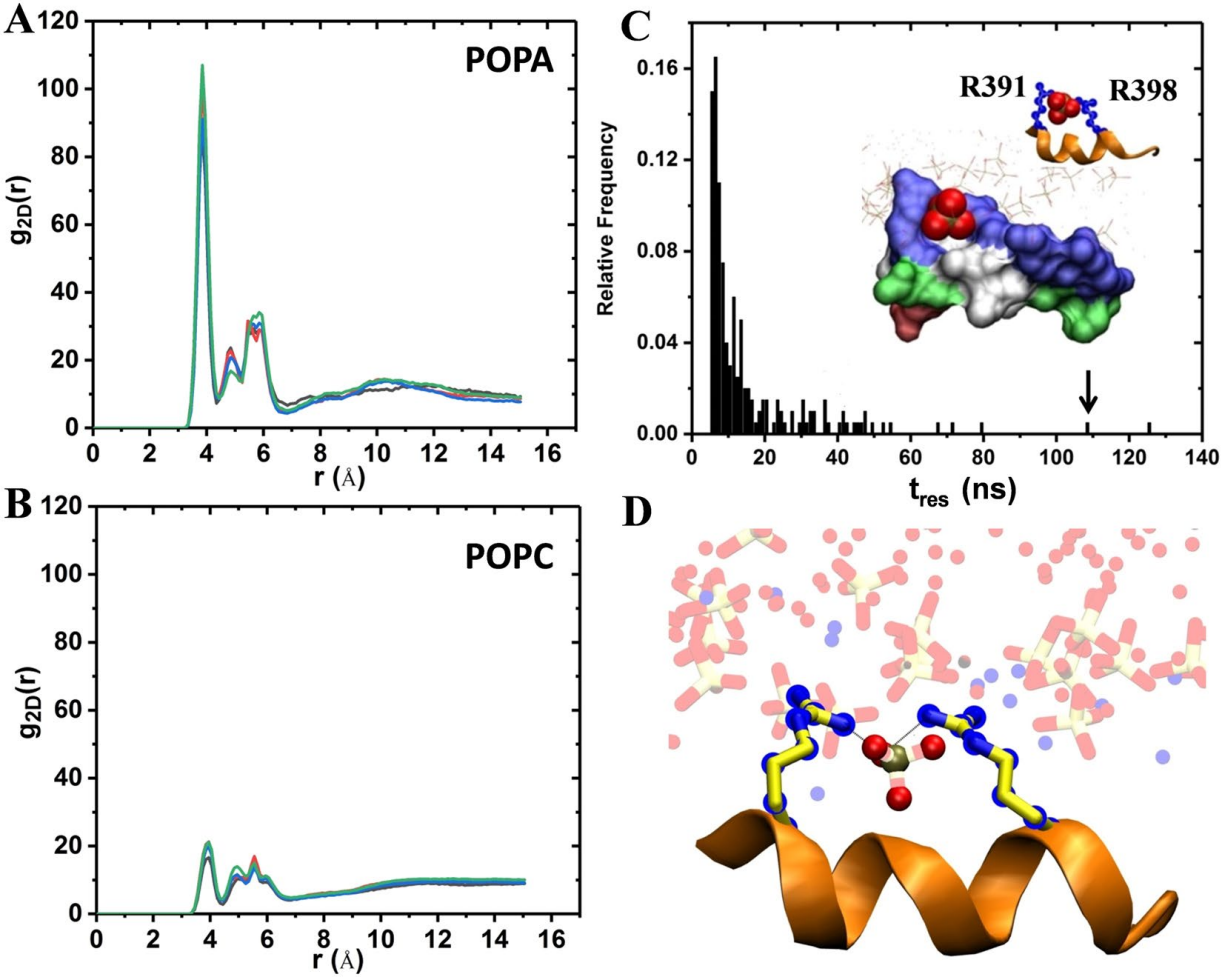
Two dimensional radial distribution function, g_2D_(r), of phosphorous atoms of PA (**A**) and PC (**B**) around specific atoms of the basic cluster residues. Nξ atom of K399 and Nδ atom of R391, R398 and R401 were used. The g_2D_(r) is averaged over all four peptides in the cRaf simulation, *cRaf* ^αC-*Pept*^. The different colors show convergence using time-blocks 600–700 ns (black), 700–800 ns (red), 800–900 ns (blue), and 900 to 1000 ns (green). (**C**) Histogram of t_res_ (residence time of PA around individual residues of the cRaf basic cluster). *t*_*res*_ is calculated using Eq. 1. A surface “groove” formed by basic cluster residues e.g. R391 and R398 is shown in surface representation with the following color scheme: blue (+), red (−) and white (all hydrophobic) and green (all other amino acids). The phosphate of POPA is shown in red (oxygen) and tan (phosphorous) vdW spheres. (**D**) Multivalent salt-bridge interaction between a single PA lipid and two residues (R391 and R398) from the basic cluster.

PA clusters around the R.RKTR motif of *cRaf* ^αC-*Pept*^ as observed from a 2D radial pair distribution function that shows a primary peak around ~4Å and secondary and tertiary peaks around ~4.8Å and ~5.7Å (Fig. 5A,B). Though only 12% of PA is present in the simulation, nearly 20% of the PA phospholipids are found near the R.RKTR motif (Fig. S4). Similar results are obtained for *mTOR*^*FRB*-*pept*^(Fig. S5). Overall, we observe that the polybasic cluster interacts specifically with PA (Fig. 5). We observed surface “grooves” formed by multivalent interactions between PA and positively charged residues (Fig. 5C). These surface grooves are formed by the polybasic residues that engage the phosphate group of PA stably and for much longer times (Fig. 5C, Table S3). This is not completely unexpected given the complementarity between the R.RKTR motif and anionic phospholipid, PA.

Next, we focused our attention on the dynamics of specific PA molecules that interact with the residues in R.RKTR. A recent study reported a range of 50 to 100 ns as the residence time of POPA near a charged motif in an amphipathic helix of a protein^67^. We observe ~15 binding events with t_res_ ≥ 40 ns, with the maximum residence time of a PA molecule, ${t}_{res}^{{\rm{\max }}}$, is 125 ns near R398 (Figs 5C and S6). This suggests that PA’s dynamics is slowed down upon binding to R.RKTR. We must note that the values of the residence time are sensitive to the Δt (10 ps in this case) and the actual residence time in some cases will be >100 ns. As an example, for one of the peptides in *cRaf* ^αC-*Pept*^, a single PA phospholipid remained bound to R398 for >300 ns with a Δt = 50 ps (Fig. S6). Note, the aggregate residence time of PA near R398 is >500 ns (Fig. S6).

Owing to the suggested PA-specificity of the αC-helix that harbors the R.RKTR motif, combined with the available knowledge that the αC-helix modulates Raf kinase function^14^ and that the dimerization of Raf involves the RKTR motif^68^, we reasoned that the membrane-interacting sequence motif of cRaf may play a functional role. We investigated this in the next section.

### αC-helix, N-terminal acidic region (NtA) and activation segment (AS) are membrane-interacting regions of cRaf kinase domain

We studied the membrane interaction properties of the full-length kinase domain of cRaf with and without tK-CAAX, $cRa{f}_{tk-CAAX}^{KinaseD}$ and *cRaf*^*KinaseD*^, respectively. The former construct is biologically relevant as it localizes Raf on the plasma membrane in a Ras-independent manner^16^ (see introduction & methods). Despite the absence of tK-CAAX, once placed on the membrane surface, Raf remains bound to the bilayer throughout the simulation in *cRaf* ^*KinaseD*^. Therefore, we merged the *cRaf* ^*KinaseD*^ and $cRa{f}_{tk-CAAX}^{KinaseD}$ trajectories for further analysis.

**Figure 6:**
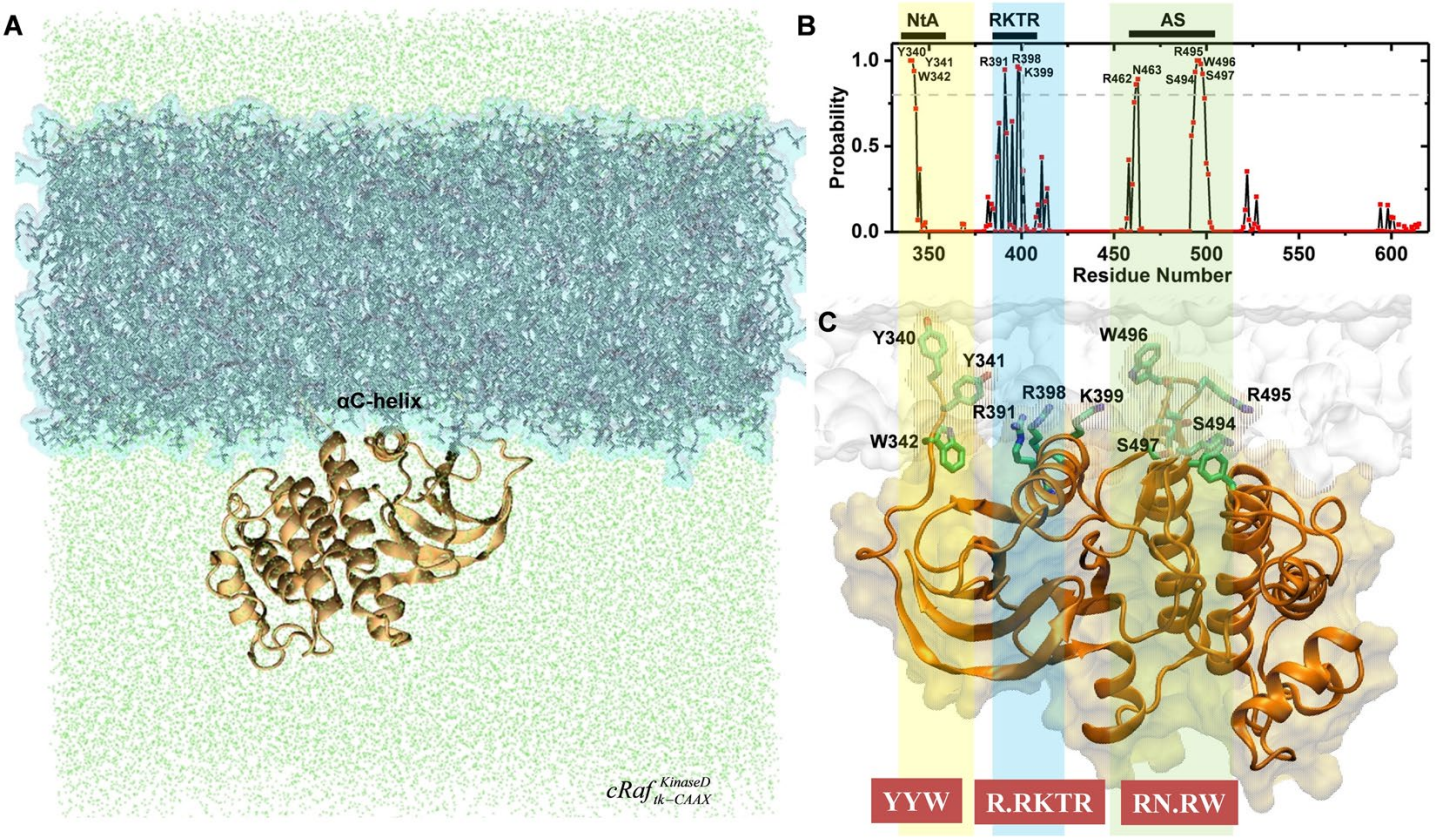
(**A**) Last snapshot of cRaf kinase domain (orange ribbons) bound to the PA/PC membrane (blue surface) from the simulation $cRa{f}_{tk-CAAX}^{KinaseD}$. Only the kinase domain is shown. Water is shown as green dots. αC-helix from the N-lobe of cRaf kinase domain is labeled. **(B)** Probability of the residue contact with the lipids in *cRaf* ^*KinaseD*^ plus $cRa{f}_{tk-CAAX}^{KinaseD}$. Cutoff criteria are same as in Fig. 4. Three regions are in direct contact with membrane phospholipids, N-terminal acidic region (NtA, yellow); the basic cluster (blue); and the activation segment (AS, green). (**C**) A snapshot from the $cRa{f}_{tk-CAAX}^{KinaseD}$ simulation with membrane-interacting residues shown as sticks (C: green, N: blue; O: red). Protein is in orange and membrane in white surface representation. An extended membrane-interacting sequence code is highlighted in the brown box.

Residues from the three regions of the catalytic kinase domain of cRaf made direct contact with the membrane for >80% of the simulation length. These are: (1) Y340, Y341, W342 at the NtA, (2) the R.RKTR motif of the αC-helix, and (3) R462, N463, S494, R495, W496 and S497 at the AS (Fig. 6). We observe that a combination of hydrophobic and aromatic residues from NtA and positive and aromatic/hydrophobic residues from AS contribute towards the membrane binding. These residues resemble the membrane-interacting residues from known membrane-binding domains (Table 1). Specifically, the NtA residues resemble PKCδ’s and AS’s resemble PKCα’s, while the R.RKTR motif resembles the KA1 domain from MARK/PAR1 kinase (Table 1, section 1). Together, the three lipid-binding regions of cRaf:KinaseD give rise to an extended membrane-interacting surface (Fig. 6C, brown boxes). Quite interestingly, R401 of the R.RKTR motif interacted only for ~35% time with the membrane in the full-length KinaseD and remained out of contact for the remaining 65%. This is interesting because R401 of RKTR motif plays a key role in Raf dimerization^10^.

The αC-helix sits in an orientation perpendicular to the membrane normal, as observed in *cRaf* ^αC-*Pept*^(Fig. S3). In kinases, a set of specific hydrophobic residues aligned together results in the formation of a regulatory or R-spine. In cRaf, F408, L397, F487 and H466 are involved in the formation of the R-spine. In our simulation, we observe R-spine residues aligned in a linear manner throughout the simulation. Additionally, we observe that W342, an NtA residue which is conserved among all Raf isoforms, extends the cRaf’s R-spine. The extended R-spine remains stable as well. A previous study suggested a similar Trp-mediated R-spine extension in bRaf dimers, though it was in the absence of membrane^35^. Since W342 interacts with the membrane throughout the simulation this suggests that the R-spine in cRaf is likely to adopt a preferred orientation with respect to the membrane plane.

**Figure 7:**
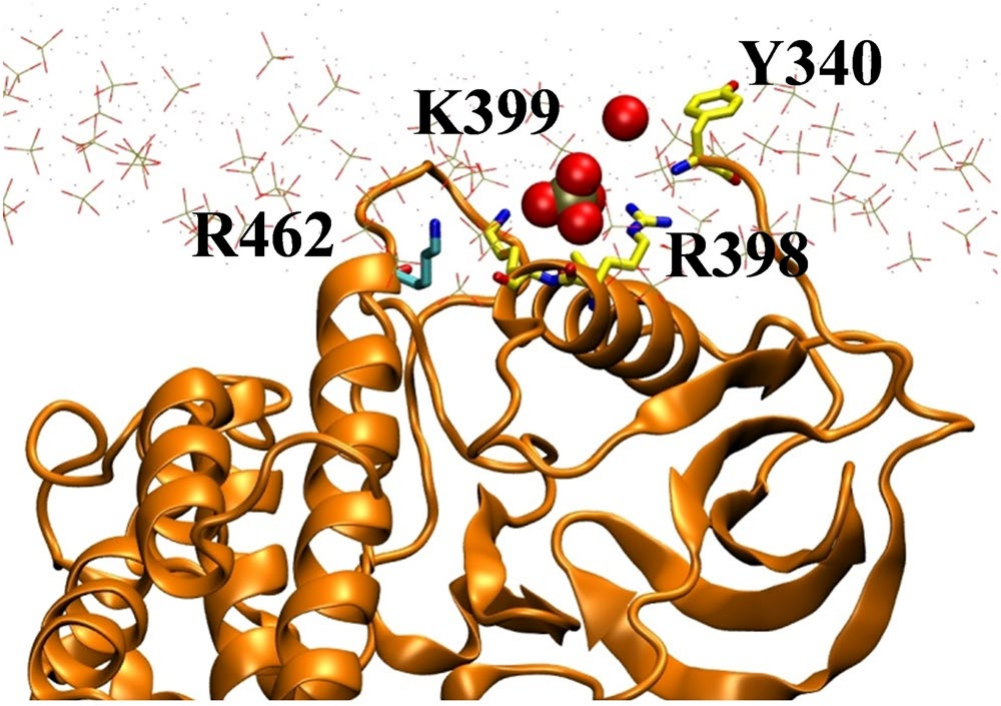
PA lipid shown in vdW spheres (red:oxygen and tan:phosphorous) with ${t}_{res}^{{\rm{\max }}}$ = 125 ns for R398 forms multivalent salt bridges with protein residues from three different regions of the cRaf:KinaseD including residues Y340 (NtA), R398 and K399 (basic cluster) and R462 (AS). The kinase domain is in orange ribbon, membrane is shown as lines with O: red, P: tan, N: blue and selected residues shown as sticks (C:cyan).

PA lipids around the R.RKTR motif show a high residence of t_res_ > 100 ns (Fig. S6). This is similar to our results from *cRaf* ^αC-*Pept*^ where the PA lipids showed longer dwell time near the polybasic cluster. In addition, residues from NtA (Y340; ${t}_{res}^{{\rm{\max }}}$ = 21 ns) and AS (R462; ${t}_{res}^{{\rm{\max }}}$ = 15 ns) also hold onto PAs with relatively longer dwell times (Fig. S6). In fact, a single PA lipid is shared among the residues belonging to the three distinct regions (Fig. 7). For example, the residues R398 and K399 (polybasic cluster) and Y340 (NtA) interact with a PA for ~152 ns (Fig. 7). Afterwards, while maintaining the interaction with the polybasic cluster residue(s), the same PA lipid interacts with the activation segment (R426).

Therefore, we demonstrate that an extended membrane-interacting surface on the kinase domain of cRaf is formed by positively charged, aromatic and hydrophobic residues contributed by the three distinct regions (NtA, αC and AS). A previous study^29^ proposed that lipid-interaction of cRaf’s PA-interacting region is driven not just by electrostatics. Our observation of additional regions, NtA and AS, making direct contact with the membrane in addition to the αC-helix (R.RKTR motif) may provide a possible explanation for this observation. This is because interaction with multiple regions especially those containing key phosphorylation sites (NtA and AS, see discussion) makes the KinaseD-membrane interaction non-random.

## Discussion

Some studies suggested a putative interaction between the phosphatidic acid (PA) and the kinase domain of the cRaf and mTOR kinases^29–31^. Such an interaction may either involve sequestration of PA by the protein or a direct interaction between protein and membrane. Studies indicated a possibility for the latter^29–31^. Based upon this, we first performed a comparative sequence and structural analysis on the known membrane-interacting domains such as C1, C2, PH and the putative PA-interacting regions of cRaf and mTOR (Figs 2 and 3). We observe a putative membrane-interacting sequence motif, RxEx^4^R(K)K(R)T(L)(Q)R conserved in Raf-like kinases across diverse organisms (Fig. 3). Our results showed the presence of a polybasic cluster in the suggested PA-interacting region of cRaf (R.RKTR) and mTOR (RRISK) kinase domains, that resembles the membrane-interacting residues from known membrane-binding domains (Fig. 3, Table 1). We thus hypothesized that the kinase domain of cRaf and mTOR has membrane-interacting regions. To investigate this, we performed MD simulations on the potential PA-interacting region of cRaf and mTOR (*cRaf*^αC-*Pept*^ and *mTOR*^*FRB*-*pept*^, respectively) in an explicit PA (12%) and PC (88%) membrane bilayer model as a proof-of-principle (Fig. 1). *cRaf* ^αC-*Pept*^ and *mTOR*^*FRB*-*pept*^ remain stably bound to the PA/PC membrane throughout the simulation, and the polybasic clusters, R.RKTR of cRaf and RRISK in mTOR were found to be the membrane-binding residues (Fig. 4). In cRaf, PA clustered around the R.RKTR motif and PA lipids interacted with the polybasic cluster with larger dwell times (Fig. 5). These interactions were stabilized by multivalent salt bridges between a PA lipid and multiple charged residues (Fig. 5C,D, Table S3). Our results from *cRaf* ^αC-*Pept*^ simulations thus support our hypothesis that cRaf:KinaseD interacts stably with membrane via its R.RKTR motif.

In order to gain insight into kinaseD-lipid interaction in a more physiologically relevant context, we performed MD simulations of the full-length kinase domain of cRaf with and without tK-CAAX embedded in the same PA/PC membrane ($cRa{f}_{tk-CAAX}^{KinaseD}$ and *cRaf* ^*KinaseD*^; see methods). Attaching tK-CAAX to cRaf mimics an auto-inhibition released state of Raf on the membrane^8,9,16^. Our results revealed that three distinct regions, namely NtA, αC-helix, and AS interact directly and stably with the membrane. Together, the three regions contributed towards the formation of an extended membrane-interacting surface involving a combination of aromatic, hydrophobic and positively charged residues: YYW..R.RKTR..RN.RW. We termed this extended surface because it is comprised of three different groups of the membrane-interacting residues in known membrane-binding domains (section 1, Table 1).

The disordered AS and the NtA regions, which are engaged in the protein-membrane interaction, contain activating phosphorylatable residues that are occluded by the membrane in our simulation. And yet membrane targeting has been shown to facilitate the phosphorylation of NtA residues^34^. It is possible that upon release of auto-inhibition and recruitment of Raf to the plasma membrane, the kinase domain interacts transiently with the membrane and thereby occlude its phosphorylatable residues and αC-helix that is required for dimerization. Then, tumbling of the kinase domain on the membrane would expose some of the activating phosphorylatable residues (e.g. NtA’s) to membrane-localized kinases. Even if slight, the repulsion between the anionic membrane and negative charges introduced by phosphorylation may expose the αC-helix for dimer formation. Upon completion of the phosphorylation, the NtA and AS regions likely experience conformational changes that prime Raf protomers for dimerization. Differential conformational dynamics of a membrane interacting phosphorylated peptide versus its non-phosphorylated counterpart has been observed^69^. Quite interestingly, R401, a key residue involved in Raf dimerization^10^ and whose mutation results in dimer disruption, interacts less efficiently with the membrane in the $cRa{f}_{tk-CAAX}^{KinaseD}$ and *cRaf* ^*KinaseD*^ simulation (only 35%). Thus, we speculate that while R391, R398 and K399 act as anchor points to the membrane, R401 that lies in a flexible loop preceding the C-terminus of αC-helix, is free to participate in dimer formation.

**Figure 8:**
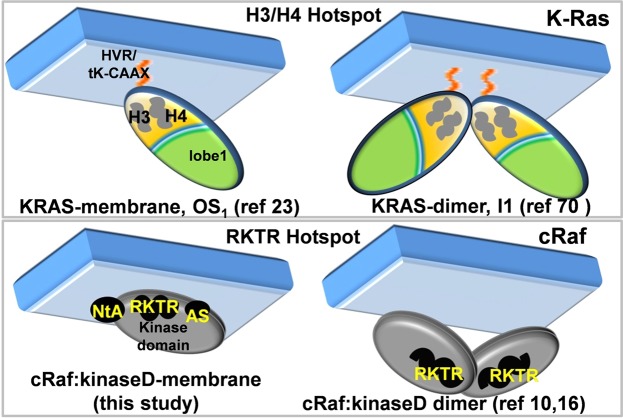
An illustration showing similarities between KRAS and cRaf. Both contain hostpot regions, helix 3 (H3) and helix 4 (H4) in KRAS and RKTR motif in cRaf kinase domain (cRaf:KinaseD). In both, the hotspot interacts with the membrane when they are monomers. Both undergo dimerization with their hotspot regions lying at the dimer interface.

Ras, upstream of Raf, is a single domain small GTPase having two lobes: lobe 1 or effector-lobe and lobe 2 or allosteric-lobe. We and others^22,23^, using computations and experiments, have shown that K-Ras adopts two distinct orientations with respect to membrane surface, one where lobe 2 directly interacts with membrane (OS_1_) and another where lobe 1 does so (OS_2_). Since lobe 1 is effector-interacting, occlusion of this surface by membrane in OS_2_ can affect its function. On the other hand, in OS_1_ helices 3 and 4 are in direct contact with the membrane, and the effector-lobe is free to interact with the downstream binding partners (Fig. 8). We also showed that the interface of one of the dominant K-Ras dimers, I1 dimer, is formed by helices 3 and 4^70^ (Fig. 8). Our results from the current work show that the same RKTR motif in cRaf is involved in membrane interaction and in dimer formation (Fig. 8). This suggests that RKTR is a hotspot similar to the helices 3 and 4 of K-Ras, and interacts with membrane when cRaf is in the monomeric state and forms an interface upon Raf dimerization.

The interaction of monomeric cRaf with membrane, therefore, appears to be an important step in the activation of Raf. The formation of this complex possibly acts as an intermediate in the activation process and (1) reduces the search space of monomeric cRaf proteins to find other protomers for dimerization, (2) facilitates the interaction of phosphorylatable regions (NtA and AS) with membrane thereby positioning them to possibly get phosphorylated by membrane-localized kinases and (3) regulates the exposure of the αC-helix via which Raf dimerizes. These are suggested by a recent study that shows membrane targeting of cRaf enhances the phosphorylation of residues in NtA^34^. An interdependence of phosphorylation and activation of kinases by membrane binding has also been shown for another protein kinase, AKT^71^. Studies have also shown that phosphorylation precedes Raf dimerization^34,35^. Interaction of cRaf:KinaseD with specific membrane phospholipids may also serve as one of the factors responsible for unmasking the kinase domain after its Ras-dependent recruitment to the plasma membrane. To our knowledge, this is the first study explicitly showing the interaction between the catalytic kinase domain of cRaf and membrane, pinpointing the key regions with possible functional implications.

## Conclusions

Previous studies suggested a possibility of interaction between the cRaf kinase domain and the PA lipids^29,30^. Molecular and atomistic details of such an interaction are missing. We present a modeled complex of the cRaf kinase domain bound to a PA/PC bilayer membrane. Our study shows R.RKTR motif is PA-specific and three distinct regions of the kinase domain of cRaf, the regulatory αC-helix, N-terminal acidic region (NtA) and activation segment (AS) interact directly with the membrane. The three regions, together, form an extended membrane-interacting surface that resembles the membrane-interacting residues from well-known membrane-binding domains. Activating phosphorylatable residues of Raf form stable contact with the membrane. We propose that while Raf is recruited to the plasma membrane primarily via its N-terminal domains, the catalytic kinase domain may transiently interact with the membrane and possibly modulate Raf activation process.

## Supplementary information

Supplementary Information to Publish
