# Supplementary material for: Three distinct regions of cRaf kinase domain interact with membrane
Source: Sci Rep. 2019 Feb 14;9:2057. doi: 10.1038/s41598-019-38770-w (PMC6375958; doi:10.1038/s41598-019-38770-w)
Supplement: Supplementary file 1 — Supplementary Information to Publish [file 41598_2019_38770_MOESM1_ESM.pdf]

Three distinct regions of cRaf kinase domain interact with membrane

Priyanka Prakash*, John F Hancock, Alemayehu A Gorfe

McGovern Medical School, University of Texas Health Science Center at Houston, Department of Integrative Biology and Pharmacology, 6431 Fannin St., Houston, Texas 77030

*Corresponding author: priyanka.p.srivastava@uth.tmc.edu

Keywords: Ras, Raf, protein-membrane interaction, dynamics, membrane-orientation

Running Title: Raf kinase domain - membrane interaction

Sequence Analysis

The sequence 1 (Fig 3) was used as a query and different organisms and different kinases were selected manually. The sequences obtained were submitted to the conserved domain database (CDD) and it reveals that in addition to Raf-like kinases whose domain architecture consists of N-terminal RBD, CRD domains along with the C-terminal kinase domain; serine-threonine like kinases with different domain architectures were also obtained. Some of them contained the PAS like domain, ubiquitin-like domain and EDR1 domains. In the CDD as well as in the PFAM motif database we did not find the RxEx^{4}R(K)K(R)TR motif listed as a membrane-interacting domain. For the bRaf sequence, CDD reports RKTR as part of the dimer interface but it does not report it as an individual motif. The full-length sequences with their accession numbers are given at the end of this supplementary data file.

Supplementary Figures

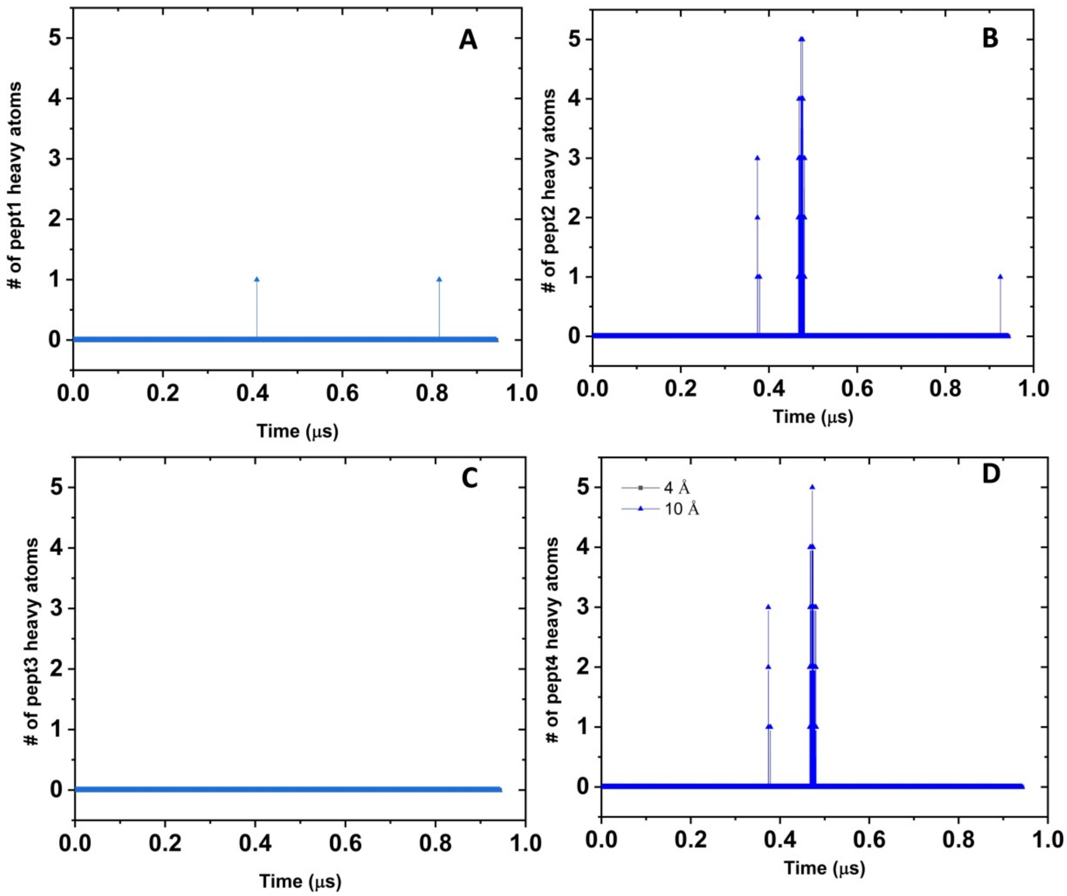

Figure S1: The number of backbone atoms of each of the four peptides within 4 (black) or 10 Å (blue) of the backbone atoms of the other three peptides in the $cRaf^{\alpha C-pept}$ simulation. No contact among the peptides is observed at smaller cutoff (4 Å). Even at larger cutoff (10 Å) only a few atoms interact and that too, only transiently.

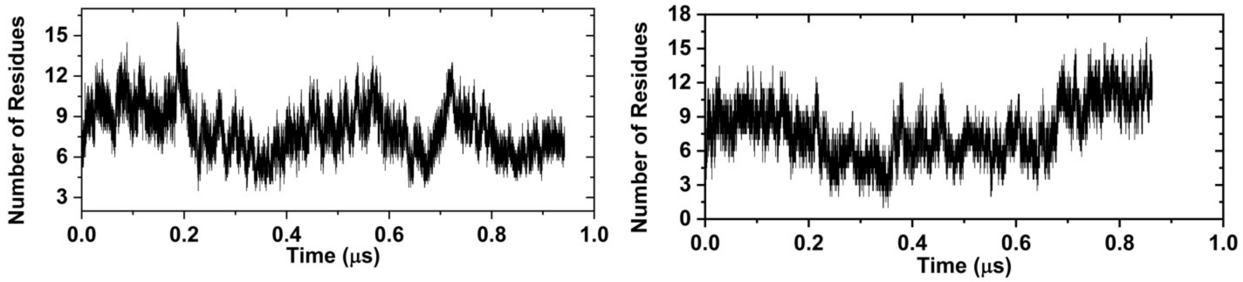

Figure S2: Average number of residues in $cRaf^{\alpha C-pept}$ (left) and $mTOR^{FRB-pept}$ (right) in contact with the lipids.

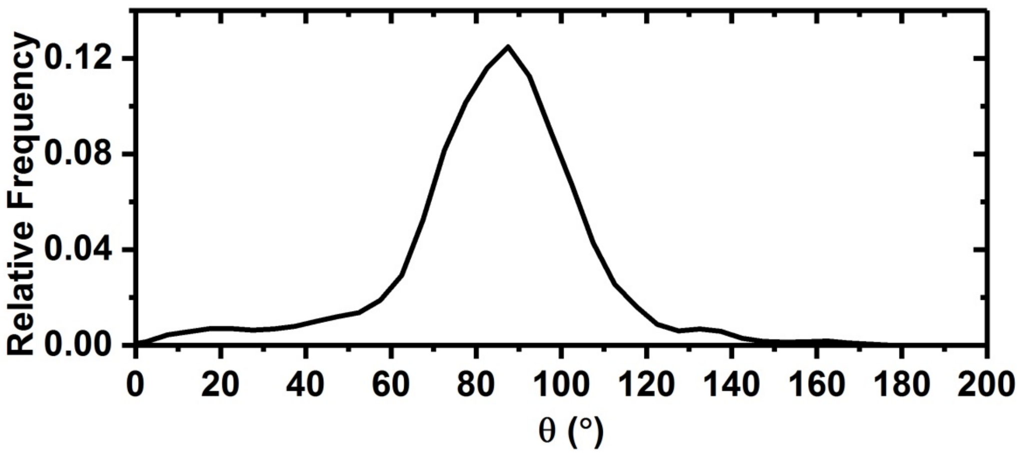

Figure S3: Relative frequency of the angle (θ) between a vector formed by the Cα atoms of residues 394 and 400 and the membrane normal averaged over all four peptides in $cRaf^{\alpha C-pept}$.

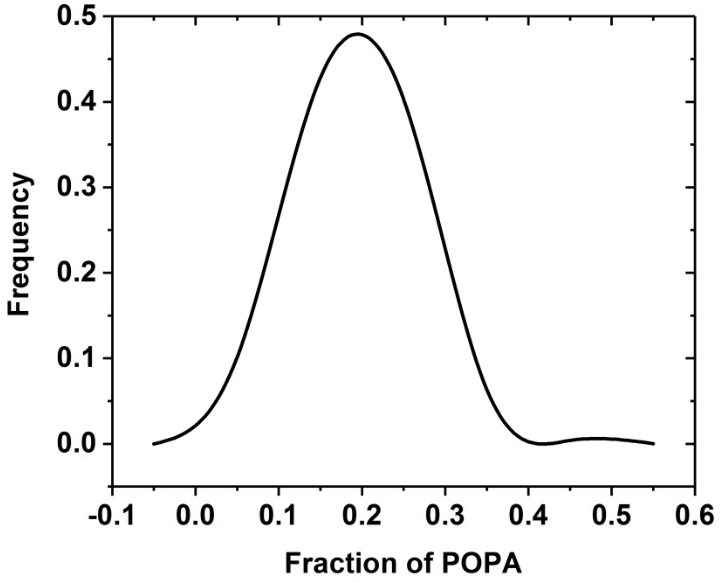

Figure S4: Fraction of POPA lipids in contact with the heavy atoms of basic cluster residues. It is calculated by taking a ratio between the P atoms of the PA lipids over the P atom of PA+PC lipids.

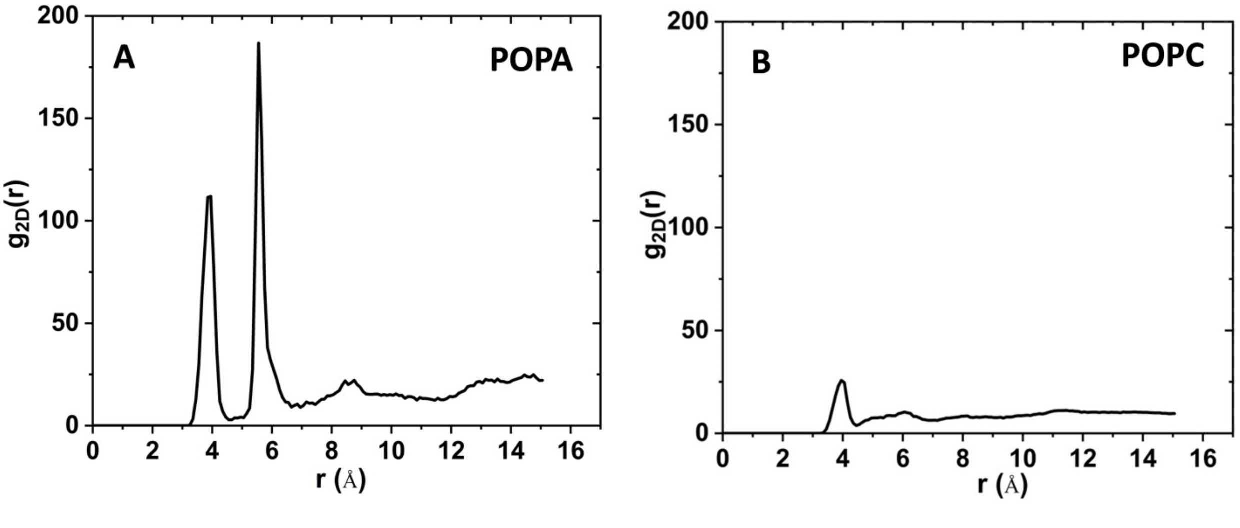

Figure S5: Two dimensional radial pair distribution function, $g_{2D}(r)$, of phosphorous atoms of PA (A) and PC (B) around specific atoms of the basic cluster residues. Nξ atom of R2109 and R2110 and Nζ atom of K2113 were used. The last 100ns of the trajectory was used for this analysis. The $g_{2D}(r)$ is shown for one of the peptides from $mTOR^{FRB-pept}$.

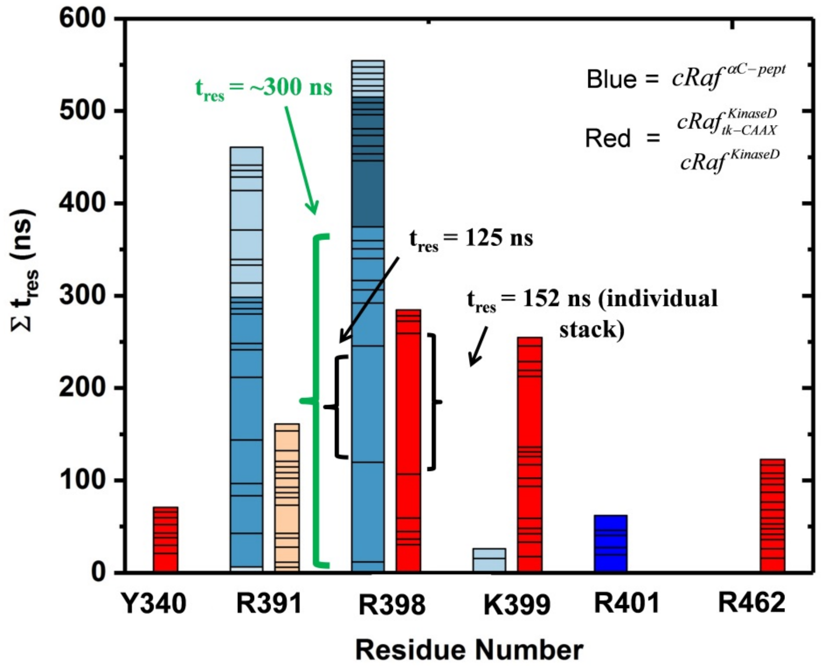

Figure S6: Aggregate residence time of residues in contact with membrane for >80% of the simulation time for one of the $cRaf^{\alpha C-pept}$ peptides (blue bars) and $cRaf^{KinaseD} + cRaf^{KinaseD}_{tk-CAAX}$ (red bars). The residence time (t_{res}) is calculated using eq 1 in the main text with a Δt = 10ps. Σt_{res} is the aggregate residence time which measures total residence time of a PA molecule around specific residues, considering only those values of t_{res} that are ≥ 5ns. Each stack in the column represents a single t_{res} value, also referred to as a stack. A contact is defined when heavy atoms of specific residue lie within a cutoff of 4Å within heavy atoms of lipid headgroups. Identical color in the stack shows identical PA lipid. Green color represents value of t_{res} with Δt = 50ps where the bracketed values represents a single stack.

Supplementary Tables

Table S1: Summary of the simulations

Simulation	Length	Remarks
$cRaf^{\alpha C-pept}$	1 µs	Four cRaf PA-interacting peptide regions; two each in top and bottom leaflets
$mTOR^{FRB-pept}$	1 µs	Two mTOR PA-interacting peptide regions; two each in top and bottom leaflets
$cRaf^{KinaseD}$	500 ns	Full-length kinase domain of cRaf (residues 340 to 615)
$cRaf^{KinaseD}_{tk-CAAX}$	500ns	Full-length kinase domain of cRaf plus the C-terminal domain of cRaf (residues 615 to 648) tethered to the plasma membrane via a 17 amino acid residue stretch from the C-terminal of KRAS (tK-CAAX)

Table S2: Summary of the sequences in Fig 3.

Seq #	Region Name from CDD	Protein	Organism
1	PKc_like	cRaf	Human
2	STKc_Raf	Serine/threonine-protein kinase B-Raf-like	Human
3	Pkinase	Serine/threonine protein kinase a-raf	Takifugu rubripes
4	PKc_like	v-raf murine sarcoma viral oncogene B1-like protein	Rattus Norvegicus
5	STKc_Raf	Raf homolog serine/threonine protein kinase phl-like isoform X1	Limulus polyphemus
6	PK_KSR	Kinase Suppressor of Ras 1	Drosophila Obscura
7	STKc_MAP3K-like	Serine/threonine-protein kinase CTR-1 like isoform X2	Physcomitrella patens
8	-	TKL/DRK protein kinase	Phytophthora Parasitica
9	STKc_MAP3K-like	CTR-1	Spinacia Oleracea
10	STKc_MAP3K-like	Serine/threonine-protein kinase EDR1	Amborella Trichopoda
11	STKc_MAP3K-like	CTR1-like kinase kinase kinase	Brassica Juncea
12	STKc_MAP3K-like	Serine/threonine-protein kinase EDR1	Oryza Sativa
13	STKc_MAP3K-like	Protein kinase domain superfamily protein	Zea Mays
14	-	TKL/DRK protein kinase	Pythium insidiosum
15	-	Tyrosine kinase	Handroanthus impetiginosus
16	STKc_MAP3K-like	Probable serine-theronine protein kinase SIS8 isoform X1	Zea Mays
17	STKc_MAP3K-like	Tyrosine kinase family protein isoform 3	Theobroma Cacao
18	-	PAS-domain containing protein tyrosine kinase	Trofolium Pratense
19	STKc_MAP3K-like	3-phosphoinositide-dependent protein kinase B-like	Momordica Charantia
20	STKc_MAP3K-like	Serine/threonine protein kinase STY17 isoform X1	Arachis Ipaensis
21	STKc_MAP3K-like	Serine/threonine protein kinase STY46 isoform X2	Arachis Duranensis
22	STKc_MAP3K-like	Dual specificity protein kinase splA isoform X2	Arachis Ipaensis
23	STKc_MAP3K-like	Dual specificty protein kinase shkD-like	Aegilops tauschii
24	STKc_MAP3K-like	Mitogen activated protein kinase kinase kinase 13 isoform X4	Capsella Rubella

Table S3: Hydrogen bonds (distance criteria 3.5 Å and angle 30°) between PA lipids and basic cluster residues that is stable for more than 50ns. The different colors represent four different peptides in the $cRaf^{\alpha C-pept}$ simulation.

Residue:Atom	Time (ns)
R398:NH2	81
R401:NH2	76
R401:NH2	176
R391:NH1	96
R391:NH2	118
K399:NZ	73
R401:NH1	139
R398:NE	76
R398:NH1	187
R398:NH2	381
K399:NZ	389
R401:NH1	170
R401:NH2	135
R391:NE	120
R391:NH1	198
R391:NH2	291
R398:NE	274
R398:NH1	58
R398:NH2	400

Full sequences in the FASTA format are given below along with their accession numbers. The region that is shown in Fig 3 of the main text is highlighted (red).

>pdb|3OMV|A Chain A, Crystal Structure Of C-Raf (Raf-1)
MDRGSHHHHHHGTQEKNKIRPRGQRDSSYYWEIEASEVMLSTRIGSGSFGTVYKGKWHGDVAVKILKVVD
PTPEQFQA**FRNEVAVLRKTRHVNILLFMGYMTKDNLAIVTQWCEG**SSLYKHLHVQETKFQMFQLIDIARQ
TAQGMDYLHAKNIIHRDMKSNNIFLHEGLTVKIGDFGLATVKSRWSGSQQVEQPTGSVLWMAPEVIRMQD
NNPFSFQSDVYSYGIVLYELMTGELPYSHINNRDQIIFMVGRGYASPDLSKLYKNCPKAMKRLVADCVKK
VKEERPLFPQILSSIELLQHSLPKINR
>pdb|4H58|A Chain A, Serine/threonine-protein kinase B-raf
DDWEIPDGQITVGQRIGSGSFGTVYKGKWHGDVAVKMLNVTAPTPQQLQA**FKNEVGVLRKTRHVNILLFM**
GYSTKPQLAIVTQWCEGSSLYHHLHIIETKFEMIKLIDIARQTAQGMDYLHAKSIIHRDLKSNNIFLHED
LTVKIGDFGLATVKSRWSGSHQFEQLSGSILWMAPEVIRMQDKNPYSFQSDVYAFGIVLYELMTGQLPYS
NINNRDQIIFMVGRGYLSPDLSKVRSNCPKAMKRLMAECLKKKRDERPLFPQILASIELLARSLP
>NP_001033077.1 serine/threonine-protein kinase A-Raf [Takifugu rubripes]
MSSTTSYSSSGETSPEDIPRGGGTIRVYLPNKQRTVVNVRQGQTVHESLDKALKVRGLNQDCCAVFRLLE
GRKRLTDWDTDITPLVGEELLVEVLDDIPLTMHNFVRKTFFKLAYCDFCHKFLFNGFRCQTCGYKFHQHC
SSKVPTVCLDMGTAKRVGVDEYPQILLSENSPSQSNLTPEPAGMDLSPTPAFHFPMPGGDGQSLQRHRST
STPNVHMVSTVGPAGASIIEEVIKFNNILGPEPSPKLSTSPPSSFGSPGRRPPKSPSEQKERKPSSSDDK
KKVHRGGYRDSSYYWEVHPREVTIQKRIGTGSFGTVFKGKWHGDVAVKILKVTEPTPEQLQA**FKNEMQVL**
RKTRHVNILLFMGYMTKPNFAIITQWCEGSSLYRHLHVTETKFDTMRRIDVARQTAQGMDYLHAKNIIHR
DLKSNNIFLHEGWTVKIGDFGLATVKSRWSGSKQVEQPSGSILWMAPEVIRMQDSNPYTFQSDVYAYGVV
LFELMSGSLPYSNINNRDQIIFMVGRGYLSPDLTKIFSTSPKSMKRLIVDCLKFKRDERPLFPQVRCTAP
KCHGQENLDFTQN
>AAK32708.1 v-raf murine sarcoma viral oncogene B1-like protein, partial [Rattus norvegicus]
LNVTAPTPQQLQA**FKNEVGVLRKTRHVNILLFMGYSTKPQLAIVTQWCEG**SSLYHHLHIIETKFEMIKLI
DIARQTAQGMDYLHAKSIIHRDLKSNNIFLHEDLTVKIGDFGLATVKSRWSGSHQFEQLSGSILWMAPEV
IRMQDKNPYSFQSDVYAFGIVLYELMTGQLPYSNINNRDQ
>XP_013777571.1 raf homolog serine/threonine-protein kinase phl-like isoform X1 [Limulus polyphemus]
MAVAVPKFNLDSPSESLEKSLDHEENGTIHDELQNIRSMIRLTRGNLEALNAKFAQYQHPPSMYITEFED
LNSKLNEFQLQEQRLVDQLSNGSNGRDSPEQSDPDYDESRMYPEIATPRETLTSNSSQASLSSCSTGTLP
DGTRLTPKSPLKVVRAFLPYQQRSTVQVRPGQTIQEALSKAMERRHLTPHMCVVYRCNPRVRIEWDTDIT
SLEGEEITVEIRERFPITTSISHNFARKTFFSLAFCECCRGILFHGFRCQTCGYRFHQRCAANVPTLCQP
LRVDHIYKQLLAMNNTASNMLQTTGDSYSPYASNYQYQRQPAVTSPPKNVPRSHPPPLGQRERSTSAPNV
CYNTVNHDMTLEEFALRLRSQNVPGVSSPLTPRYEIFSRFHTHKGSPTHSPVPKRRQLELPCPGGSPGSL
ALPYQQSSASTNHSPSSSPTRTQSAQGSPTNIHKPWRPRARSADESSKKVQRTTRESIEDWEIPANEILT
GPRIGSGSFGTVYRAHWHGPVALKKLNVTDPTAAQLQA**FKNEVAVLRKTRHVNILLFMGWVSKPHLTIVT**
QWCEGSSLYKHLYVQECKFEMVELINIARQTAQGMDYLHAKNIIHRDLKSNNIFLHEDWTVKIGDFGLAT
VKTRWSGSEQFNQPTGSILWMAPEVIRMKDSHPYSFQSDVYAFGIVLYELIAGQLPYARINNRDQILFMV
GHGYLRPDLSNARSDTPKALIRLIEDCVKFNREERPLFRQILASLELLARSLPKIHRSTSEPTLNRTHLQ
SEDFTYICASPKTPSQFGAFPFF
>XP_022223911.1 kinase suppressor of Ras 1 [Drosophila obscura]
MSSAAQLTTSGVSNNNATDSSSNANANESGSSDSNLIIIQDMIDLSANHLEGLRTQCATSSMLTQQEIRC
LESKLVRYFSELLLTKIRLNERIPANGLLPHATGNELKQWLRVVGLSQESLSVCLTRLTTLEQTLQLSDE
ELKQMLAENPNQREEELRRLTRAMQNLRKCMDTLEANTASNNDPEQWHWDSWDRPIHIHRGSVGNIGLGL
GLNTASPRAHHRQHGTKGKSGSNASTSRSGRQSPTSMPATTTEDMTSSTQGSQMTLTLTPSPPNSPFTPS
SGTAMSSSLNGTPQRSRGSGTPPPARKHKTTLFNQAAGAGDIEPPAPPRNRLPTDPSPDSHSSASSEIFG
ESSNSNPLASIGNSSNVLLVPCSPGVGPVGMGHTIKHRFKKKALGLMATCTLCQKQVFSSWLKCIDCKFI
CHKNCAPHVPPSCGLPREYVDEFRHIKEQQGGYSTLPHVHASSKGSPLVKKSTLHKPLQQQHGDSSSPSS
SCTNSTPSSPALFHQRERERDQQDQAGSYGGSSAGGGSSSSANLLLTPSLTKHHQQSQFNFPNVTITSS
SGGGSGGNGGNASHNVQTLISNENLLEYPAAPPSVANSMNGGGLIDSLVNSSNGHISSLIHSQVSNASNA
TASLVNSTTTTSTTSSFFPRKLSTAGVDKRTPFTSEYTDTHKSNDSDKTVSLTGSASTDSDRTPVRLDST

EDADSGQWRQNSISLKEWDIPYGDLRLLERIGQGRFGTVHRALWHGDVAVKLLNEDYLQDEHMLET**FRNE**
VANFKKTRHENLVLFMGACMNPPYLAIVTSLCKGNTLFTYIHQRREKFAMNRTLVIAQQIAQGMGYLHAR
DIIHKDLRTKNIFIENGKVIITDFGLFSSTKLLYCDIGLGVPHNWLCYMAPELIRALMPEKPKGECLQFT
YYSDVYSFGTVWYELICGEFTFKDQPAESIIWQVGRGMKQSLANLQSGREVKDLLMLCWAYEKEQRPRFA
QLLYKLEQLPKKRLARSPSHPVNLSRSAESVF
>XP_024390551.1 serine/threonine-protein kinase CTR1-like isoform X2
[Physcomitrella patens]
MSSGDDDARVRELRRGHSAQSSRERFHQEQGQVLQQPPPQQRRSVLAMPLAQYSRPSSFSGSGGGGSGPS
GSLFGRAGSTKQQSTVQNPSQPCVSIDLRGNITSWNRAAEQLYQFKEGEVMGRNLVDLIVPPNAKTAALD
TIMRVGQGEVWIGQFSCVRRDNAILNTVVTQKPILDDDQKISGISVCTEPYSMPELMARNYFDEGTGKIT
DENNVGDAVHLPNKSALHHPSDGDAPGKNLLKLGRSSVEQLLRKLHIGAGMRREEEDAGPRWPPERQNSW
ESEDPNDFLQERERMSHGSVAGRMHNDLYRTGDLSNVDGFALPLVAVGHGGGPYIMGGEGEHEEFGGFQG
SSAESTAWNAWIGPSGGVNFQQAEEDFQLQLALALRVAAEAAAVDDPDLSANKRGPLGSARLVPGVSRVE
STAYRYWVSNCLGYEDRIEDGFYEIWGMSPYVWSMCTDSNELGRMPPLESLRSVNPAEAEFEVVLVDRNG
DPHLRELEDKAVSLAYESQEVLDLAAKLAQMVAIQMGGSAVSDEALAETWRTNTSKMTLLLGSLVLPIGM
LKCGLGRHRALLFKVMADSVGLPCRLVRGSSYCGKEDDAMVVVKCGDDREWMVDLLVKPGQILAPDSRLA
APPAVIASPLQFERQGPFGGSSVSVLHYSRSEVGNKEAGSGLKVGGASDATLSVPSTTAVPTVPIKIDID
QGSLSSPRGPSQNMGPRRGGIPSPRGNVAPQPFDFTPLQFDQRTSVAQIAREREMEKENSSPMENHLRGG
QYDGSQSSQPSQNQDAGRTRHSEVGSGQSDDVAGRVHPPERGASSSQEEESSSKYSRDRLVENAIEFARE
FEIPWEDLIIGERIGQGSYGKVYRADWQGSDVAVKVFLDQDLKVEALEE**FKREVAIMRRLRHPNVVLFMG**
AVTVPPNLSIITEFCPRGSLYRLLHRPNRELDERRRLRMALDVVKGMNYLHRSSPPIVHRDLKSPNLLVD
KNWTVKVCDFGLSRLKHNTFLTSKSSAGTPEWMAPEVLRNELSDEKSDVYSFGVILWELATLQQPWAGMN
PIQVVGAVGFQHRRLPIPESIDSNVSNIIKACWRMDPRSRPTFSDIMQELKPLTRPAMSQLGGTPKSGLS
DRDL
>ETO78133.1 TKL/DRK protein kinase [Phytophthora parasitica P1976]
MEVPHTFVRTVKAQDLRFSQVDSVLHDIKTKNSPTWVIFSFADQREKRNTLKVLASGHGSISDQSKWPSE
LFSPNALCYGLTSIDCYDENSAPTNVLSFCWKGRNLPIYLRTKYAEFNQVIRKHMGAVAHVTLHNPEELM
DGSVFLQTPRRRPRSSSRRFVTSDIEFTPEVTGTVQDIRSDTSPFNWAVCGYDNSDLESSTRRMIVVEKG
MTGLYALKGIGGITSVLQEGSTMYIYLRVDVPLHRGTERIVSKYVLVTWQGVERSYSGGTSDDEVELWFG
PSSNGERSPQRRDSFASSSKLQRAVTAHVHGSEIYRVFPHHVHFFASTQSEISEEAIRERIRRAVDNDCM
LLRVVCVQASGDTQAPVCLEIPFDATVGVLRQEIASNIGIPKDRQRIVWIRQTDDLAKDSQLSTALMLED
ENAGIRKDIGLAHGDKIHVDDKDNGENSVLAKLFEQINSGAAAVSLSAERRHEVQRGVEAREAELRKILS
MTHYLIRRKANEDLKPVARLAGVVEEAVNREKKETDSASPTSQHKVIHRADSAASLTGSISEELAKEDVS
ALQEHARVLESQNQYLEIPYESIHILSGKENELGCGKAATVYRGLWMNRNSAAEVAVKSFRYARLTDKIL
GD**YRQEVALLRKLKHPNIVLFIGACTDPKLMILTEYC**SRKSLFEVIHSNNFETIPWKFKVRMMLDAARGI
QYLHSKRIIHRDIKSHNFLVDDDWRVKVADFGISKVLDTDTAFTQCGTTGWVAPEVLLDEDLGYTFKADN
WSFAIVMWEMIAGGLQNPFIGMAPIKFYNKTINAGIRPPIPDGVDSEYINLLTECWKSDAADRPSFDVIV
SRLEQMLLDLGASIDLPPTFQGGYHQAGLAPNP
>XP_021857231.1 serine/threonine-protein kinase CTR1 [Spinacia oleracea]
MEMPARRSNYTLLSQVDDHTTPPPSTVASKFSGVGGGERSLFPPSIGLQRQSSGSSFGESSLSDHYVPSI
SLPASDLDGFSYMQEERDSRKRVAIDGGGGGGGSSASKSWAQQTEESYQLQLALALRLSSEAACADDPNF
LDPMPDESSSRLSSSESAESISHRFWVNGCLSYSDTIPDGFYLIHGMDPYVWTVCADLQESGRIPSIESL
KLVNSVNESSLEVVLIDRRGDSSLKDLLNRALDISCKCVTSDDVVDQLAKLVSSRMGGVASTREEELVPI
WEEFSNGLKDCLGSVVLPVGSLSIGLCKHRALLFKVLADSIDLPCRIAKGCKYCNRNDAASCLVRFGLDR
EYLVDLVGKPGCLCEPDSLLNGPSSISISSPLRFPRFRSAEPAIDFRSLAKQYFSDCQSLNLVFDRPSTG
AVVNEQDAGDPMYLKQLGRKLPDVNNPIPNDQVHSQFVGHDREPQCSESSNPPQNIGRASNAIREFLPSR
FVPPSRRGTQALTDSARNASKDFRFEASQLVSSRSEAALDMDDLIISWSDLVVRDRIGAGSFGTVHRAEW
NGSEVAVKILMESDFHPERCNE**FLREVAIMKRLRHPNIVLFMGAVTEPPNLAIVTEY**LCRGSLYRLLHKS
GGREVLDERRRLSMAYDVAKGMNYLHKRNPPIVHRDLKSPNLLVDKKYTVKVCDFGLSRLKANTFLSSKS
AAGTPEWMAPEVLRDEPSNEKSDVYSFGIILWELATLQQPWNSLNPAQVVAAVGFKGKRLDIPSDVNPQI
ATIIKACWANEPWRRPSFANLMDSLRSLLKPPAPQSVRGDAHIIT
>XP_006826255.1 serine/threonine-protein kinase EDR1 [Amborella trichopoda]
MKHILRKLHIGSSHEPPRSNDPGAASPAQSCGSDHRAAVSWSPNGVPPQSPSPSSSPGGEGSAGSRLLTE
DKNDYFSSEEEFQVQLALAISASNSEFRAKDQGKAVILGSGHMQSGSSQTEDLAECLSRRYWDYDVLDYE
DKVLDGFYDIHGLSADPSSQGMPSLVDLQMTTGASSYEVVIVNRAIDPALDEMEQVARCIALDCHAPEVG
LLNCGLVQRIADLVSEHMGGPVRDANDMLARWMERSFELQATLHTNIYPIGCLKIGISRHRALLFKVLAD
TVGILCRLVKGSLYTGIDDGAVVIIKSEDQREFLVDLMGAPGTLIPADILPAKDVPMDDQRSNRNLLPRT

GYDRNRDRRTSNEVPIMRNNGILDVSSRTGKVVSQLPLPSPNGGAISMNHGNRSDGILKGVVPVNQDFQL
SSSAGATSSSKQRGDCGSSIVCDGVNEKRNVVPPTQDNMDSENLFADLNPFQKTGAGKIPAQNQVGEKKI
FEYQRRRENIYLGPGRPPLPLAWKNRNEIPRTKQQESGEGLFPRNNVDIKAANASASLMNSDASKNGELD
SSVFRTPQALGSLNSSRTGSVDNDTENTSVGNVVNMPYDSGDSSISSKVGGTELSCVGGSAGVERSYSPS
NGILDPLALGNNRPMGNPGDGIAMDNYGVVDESKEPARNRKYHIDKRKCTNDRFMEPKLTSNDQENPGPV
RRGLSRLDPMLDDVSEWEIPWEDLVVGERIGLGSYGEVYHADWNGTEVAVKKFLDQDFSGDAIEE**FRSEV**
RIMRRLRHPNVVLFMGAVTRPPNLSIVTEFLPRGSLYRLLHRPNCQIDEKRRIKMALDVAKGMNCLHSST
PTIVHRDLKSPNLLVDRSWNVKVCDFGLSRMKHNTFLSSKSTAGTPEWMAPEVLRNEPSNEKCDVYSFGI
ILWELATLRMPWSGMNPMQVVGAVGFQNRRLDIPKEVDPLVARIIWECWQTDPNLRPSFAQLASALKPLQ
QLVVSQQLDAQSSPMPQEIPVNSAP
>AAP86285.1 CTR1-like kinase kinase kinase [Brassica juncea]
MPDSVRSMKMNMKSFLKKLHITPNQPDEAADGSSIPTNKSSDVSSSPHHQSPEVKPFSGLSNWLSSVGHR
KSPSPPNSLNATNGGDDEQQQDSKEDPEVEEEYQIQLALELSAREDPEAAQIEAMKQFSLGSCAPDNSPA
ELVAYRYWNYNCLGYDDKILDGFYDLYGVLNASSAEKIPPLLDLQGTPVSDGVTWEAVLVNRSGDYNLLR
VEQMGIDIAAKTESVSSSSFVNSELVRKLAVLVGDYMGGPVVDPDSMLRAWRSLSYSLKATLGSMVLPLG
SLTIGLARHRALLFKVLCDSVGVPCRIVKGQQYTGSEDVAMNYIKTDDGREYIVDLMGDPGTLIPADAAG
LQIDYDEPVCSTSPGDNDSFHDASSTNGIESSFQENIEFPPGEHSSSTKSSKEDVAKVEKAPPVQNLSSR
PIHSFTHMRSPSWTEGVSSPAARRMKVKDVSQYMIDAAKENPRLAQKLHDVLLESGVVAPPNLFSEVYPQ
QLDATVEIKNLTEAKKEKETAQQGRHQNDLGPVRFLPPLPRLHSKADTHDQQHDHGKVVSQSDSSHSEAS
STEYARTVPAAVAAAAVVASSMVAAAAAKTANTESSTLELPAAAAATATAAAVVATAAAVSRHLELGSNS
DGDAGSGGHEPQGSGDSPHGPNSGGDRVSDRSTGDESSKSDGTLDDVSDCEILWEEITLGERIGLGSYGE
VYRGDWHGTEVAAKKFLDQDLTGEALEE**FRSEVQIMKKLRHPNIVLFMGAVTRPPNLSIITEF**LPRGSLY
RLIHRPNNQLDERRRLRMALDAARGMNYLHSCSPMIVHRDLKSPNLLVDKNWVVKVCDFGLSRMKNSTYL
SSKSTAGTAEWMAPEVLRNEPADEKCDVYSYGVILWELFTLQQPWGRMNAMQVVGAVGFQHRRLDIPDFV
DPAIAELISKCWQTDSKLRPSFAEIMVTLKKLQRPATGSNIPRPVPSSSSLSTEQEQKDC
>XP_015631594.1 serine/threonine-protein kinase EDR1 [Oryza sativa Japonica Group]
MKNLFKSKIKWQHRSNDPASSQGQGQGLPQQTPPSPSPASSPSGGPPALSVSTVSSSSPSAAATPTGAAA
AGAGGGGGGTGGEDYMLSEEEFQMQLAMALSASNSECVGDLDGEQIRKAKLISLGRGDRFAAVRDDEQTA
DALSRRYRDYNFLDYHEKVIDGFYDIFGPSMESSKQGKMPSLADLQTGIGDLGFEVIVINRAIDTTLQEM
EQVAQCILLDFPVANIAALVQRIAELVTDHMGGPVKDANDMLTRWLEKSTELRTSLHTSLLPIGCIKIGL
SRHRALLFKILADSVGIPCKLVKGSNYTGDDDDAINIIKMNEREFLVDLMAAPGTLIPSDVLSWKGNSLN
SNARLTQNPLAGSSSTTDSNLSANALPPGHKGGQLPLFSSGDWISASQSGYEKDGATTSSQASSSGTTSV
AAGSAFDSSWTLVSHGQSDDPSTSAGMSAQQKVILPGGEHPWNENINARNENIKLVSDLQGNSESINLFA
DLNPFGGREPKRTSVPLNGPDNRNNELQRRRENVVPSTRRPQQRLVMKNWSPYNDVSNNKQYNYVEDSFA
RRNIGDNAASSSQVPRPSAKNTNLNVVVRTDTPYMAAHNYDNSMAGSSAMKMTSTAGIGKVPDKVLYGDL
DKGLTNSRLGDQPPIERHKWGNSVEGRIPTGTVHNQAKEHKENFDGKQDNKKLHPDPKKSPLDRFMDTSM
PSRNPESVSPSFARSHKLDTMFDDVSECEIHWEDLVIGERIGLGSYGEVYRADWNGTEVAVKKFLDQDFY
GDALDE**FRSEVRIMRRLRHPNIVLFMGAVTRPPNLSIVSEY**LPRGSLYKILHRPNCQIDEKRRIKMALDV
AKGMNCLHISVPTIVHRDLKSPNLLVDNNWNVKVCDFGLSRLKHSTFLSSKSTAGTPEWMAPEVLRNEQS
NEKCDVYSFGVILWELATLRMPWSGMNPMQVVGAVGFQDKRLDIPKEIDPLVARIIWECWQKDPNLRPSF
AQLTSALKTVQRLVTPSHQESQSPPVPQEIWVNSSTP
>AQL09690.1 Protein kinase domain superfamily protein [Zea mays]
MKSLFKSKIRWQHRSNDPASPAAGAGGEDYISSEEEFQMQLAMALSASSNSDCAGGLDGEQIRKAKLMSL
DRFAAHRDETHTAEFLSRRYWDYNFLDYHEKVIDGFYDIFGSSIESSRQGKMPSLADLQTGIGDLGFEVI
VVNRAIDSTLQEMEQVAQCILLDFPVANIALLVQRIAELVTDNMGGPVKDANDMLTRWLEKSTELRTSLQ
TSLLPIGCIKIGLSRHRALLFKILADSVGIPCKLVKGSNYTGGDDDDAINIIKMDNEREFLVDLMAAPGA
LIPADILSWKGNSLNSNRKLGLNRTAGSSSTVDANVDPTVLPLEPKGGQLPLFSSGDWMSDIDSGYEAAE
IAASTQTSSGVTPSVSAGSVFGSSWMLVNHDQSDGPSTSAGTSSQQKVVPQSEHQRNLNRLPDLQEIPES
KYLFADLVPSGDNKSKKTSVPFKGPDHRNNELQKRRENIVPNAGRPQQRLVMKNWSPYNDVSNNKQYNYV
EDSFARRNVGNNAASSSSQMPRPAVRSNLNSGLHNDASYHNYDSIMAGTSAMKITSTAETGKVPERVLRG
DLDKGQTNYRLEDQHVIVQPPQGRLPWGNPAEGRVPMNRVQSQAKQHLENMDAKQDHKKLLPDPKKSPLD
RFMDTSLPSRNMDMRSQRLDFDDVSECEIPWEDLVIGERIGLGSYGEVYRADWNGTEVAVKKFLDQDFYG
DALDE**FRSEVRIMRRLRHPNIVLFMGAVTRPPNLSIVSEY**LPRGSLYKILHRPNCLIDEKRRIKMALDVA
KGMNCLHTSMPTIVHRDLKSPNLLVDNNWNVKVCDFGLSRLKHSTFLSSKSTAGTPEWMAPEVLRNEQSN
EKCDVYSFGVILWELATLRMPWSGMNPMQVVGAVGFQDRRLDIPKEVDPLVARIIFECWQKDPNLRPSFA
QLTSALKTVQRLVTLCHQENQSPHVQQEISVHLTP

>GAX93357.1 TKL/DRK protein kinase [Pythium insidiosum]
MDGTQVAHTFVRNVKAADVCFDQIDDVLDAIRRGKSPVWAIFCFADQQESRNTLKTIARGDDDLSDQTVW
PPELFASNALCYGLSRIDFPDEDGLASGFVSFCWKGRSLPIYLRTKYAEFNHVIRKHLQGSTHVTLQYPE
ELVEGTVWKQMPPTPRRKRSGSRRWITNEIEFAPEVLQAVKDIRSDAAPFNWLVCGYESYDPXHMQRLVV
VEKGMTGLYALKGLGGITATLKEGCTMYIYLRVDIPLHHAAERIVSKYVLVTWQGGDSQMLSDDEWGDPW
GFSASDAAPPLSSSSLRDSFVIPAPPSSSKVQRVVAAHVHGSEIYRFFPHHSSLLTSCGKLQVHFCANMT
SEITEETIRDRVRRAIDNDCMLLRVVCIQRTGDTQAPICVEVPYDATVGQLRKEITAYVGIPEARQRIVW
IRQTDEFVNELQRGIKAMLLDNDDASLRTDVGLGHGDKIHIGDSEEGGDSVLEKLVQQINTGAASVSLSA
ERRHEVQKGVEAREAELQKILVATQDKIRRRASRPDLTPPTSNTSLNGDDAHAPVHHSNHSVQSEADSLN
VGEPLSPTSEELAQEDVSNLQEQARVLEEQNEYLEIPYDSIRILSGKENELGCGKCATVYRGLWMNENGA
AEVAIKLFRYVRLTDKILGD**YTKEVAMLRKLRHPNIVLFIGACTDPKLMILTEY**CSRKSLYEVIHSNNFS
SIPWKYKVRMMLDAARGIQYLHSKRIIHRDIKSHNFLVDDDWRVKVADFGISKVLENXANAFTQCGTTGW
VAPEVLLDEDLGYTFKADNWSFAIVMWEMVAXGMQNPFIGMAPIKFYNQTVNSGIRPPFPDNVDSEYAVL
INDCWHSNPSDRPSFTEIVSRLEKIVERLGASTEMPPTFLGGYHQCLSTR
>PIN08497.1 Tyrosine kinase [Handroanthus impetiginosus]
MCTFRTTISTAMDGGEESQANLYRVLLDRFQSLEASHQKIKEQFKVLSEEKTRNTACEASLEKDVASDSE
DMTPFPGWGCLQGAYFFRSPYRSVLEYMGHAVHVCSTGSGEIVYWNSAAKKLFGYKDYEAVGRRVVELIF
DEQHLPSALTVMRTVSTGKSWTGQLPLKKRSGQNFMAMVTKTPLYEDGELVGIITVSSDANVFNGTNSGT
MRTHQDHTNEQSKVRRINFKKIQWHPDPQIVAVPQLASSVSNLASKVLLWKRGDVTCNACRPTRETEEAE
VESQDAKADRPPRAPILNSGFSMFIGRSRTDVMSSEKEGSTSEFVQPSKIAEKFFSKLQIGPISNSEKNK
DENIQQNGLEDNPRKNGTSERWYQRDSVENTSHLYDRDANYHPEDAVIGIADVAEREHLDMHMNTSVTNE
SSSTGAFSGAYQGCFEVHRPADQMPGSGFQSDRRESIANLPNIKLADSEDVSPRQSGSRETRSTGGSCGN
GPGSSPSKGDSDTHLVVDSEIRWEDLHLKEEIGQGSFAVVYRGVWNGSDVAVKVYCGNQYNEDRLLD**YKK**
EIDIMRKLRHPNVLLFMGAVCTEEKLAIVTEYMTRGSLFKTLHRNGQSLDIRRRLRMAIDVARGMNYLHH
RNPPIVHRDLKSSNLLVDKSWTVKVGDFGLSKLKNATFLTARSGRGTPQWMAPEVLRNEPSTEKSDVFSF
GVILWELMTERIPWSNLNFLQVVGVVGFMDGRLDLPENIDPQVSSLISHCWRSNPEDRPSFEEIIRKMTD
IVHAGAGVTVKTPSTP
>XP_008659085.1 probable serine/threonine-protein kinase SIS8 isoform X1 [Zea mays]
MKNFLRKLHIGDSAAGDGASSPSAPTPPPTKKGGGIEHRHASGLSGWLSSVTSRPHPPTPLSASAAPAAA
APEVEESALTTALASSVEEKRVAEEDEERARRESRKEAEMEKKEKQQAELENYHMQLALEMSVREDPEAM
QIEVAKQISLGSCPIQSSPAEVIAFRYWSFNALSYDDKILDGFYDICATGDELAMSTIPSLMDLQALPFS
HGGKTDAVLVDRALDSELVALEQKAVIMAVEFRSKKSEFVDRSLVQTLANLVSNYMGGPVIDPESMLLKY
RNMSSALKADIRSAVVPLGQLTVGLARHRALLFKVLADSLAVPCRLVKGRQYTGSDDGALNIVKFNDGRE
CIVDLMIDPGTLISSDGADLGRELEDSLVVDNQHVNKDDTSTQLLSSFSEASSSMHSSFENGSLEKGFIC
NAGHFGPYEATTAHSDNDVLGSGVSSSFDELSVSTYACENIPIIHESNTDHTMTEKSKDKSITSNNSSSC
SPPSSEIGSTPAVRRKKVKDVSEYMISAAKENPQIAERIHAVLLESGVVPPPDLFSEESKEQPKDLIVYD
TSLFQTTDEMIRTMNELESTAHDGCAGLGPSLPPDPGHELQTKIVPYRMPPDLKPVQGLGVYHKFDFRDN
INPSIPLYEPSPAPQENPLQLIKQMPVTAAAVATAAVVASSMVVAAAKSNSDIKLDVPVAAAATAAAVVA
TTAAVNQQYEYLDPGCQLLCLPSSSNKLIQKGRHDLLDDDQLETNHGQDNALEREKDLVQAPQEAERISD
RSTGTESARSEISLDKIAEFEIQWEELTLGERVGLGSFGEVYRGEWHETEVAVKKFLQQDISSDALEE**FR**
TEVGIMRRLRHPNVVLFMGAVTRVPHLSIVTEFLPRGSLFRLIHRPNNQLDQKRRLRMALDVARGMNYLH
NCTPVIVHRDLKSPNLLVDKNWVVKVCDFGLSRLKHSTFLSSRSAAGTAEWMAPEILRNEPSDEKCDVFS
YGVILWELCTLLQPWEGMNPMQVVGAVGFQQRRLDIPGGVDPAVAEIIRRCWQTDPRMRPSFSEIMATLR
PLLKNMPANQPTRKRAQQTDD
>EOY21275.1 Tyrosine kinase family protein isoform 3 [Theobroma cacao]
MPSLLDLQGTSVSDNVSWEAVLVNRAFDANLLKLEQKALQMTARLRSESLAFVSSNLVQKLAVLVSEYMG
GPVADPDNMSRAWRSLSYSLKATLGSMVLPLGSLTIGLARHRALLFKVLADSAGIPCRLVKGQQYTGSED
VAMNFVKLDDGREYIVDLMADPGTLIPSDAAASHVEDGDSFFSTSPLSRDIDSSHVASSSSGVWSLFEDN
SEFGTLEKRSRFKNFAAAGNQSDERGDLNAFVNLSGTTRSGEQSKESMDDFKTPSNMEEAPVRELPNRPN
YLYSHMRSPSWTEGVSSPAVRRMKVKDVSQYMIDAAKENPQLAQKLHDVLLESGVVAPPNLFSEIYSEQL
DTSTIEVRLPFETKDESRQGTGPQESKNQNDFGPSHCLPPLPNRKVFAKASSPCNQPEHLKPVEGLGVTY
PFDTREVIGPPVLSQSEAAPIQYARNVPVAAAAAAAAAVVASSMVVAAKKSGTDSNVELPVAAAATATAA
AVVVTSAAVTKHNERSDGDVDATGCESQGSGEREHDALGLNSEGERISDRSTGNDSSKSDVALDDVADCE
IPWEEITLGERIGLGSYGEVYRGDWHGTEVAVKKFLDQDISGESLEE**FKSEVRIMKKLRHPNVVLFMGAV**
TRPPNLSIVTEFLHRGSLYRLIHRPNNQLDERRRLRMALDAARGMNYLHNCTPVIVHRDLKSPNLLVDKN
WVVKVCDFGLSRMKHSTYLSSRSTAGTAEWMAPEVLQNELSDEKCDVYSFGVILWELCTLRQPWGGMNPM

QVVGAVGFQHRRLDIPDDIDPVIAEIIRRCWQTDPKLRPTFAEIMAALKPLQKPITSAQVPRSTASSSSA
HERGQP
>PNY12217.1 PAS domain-containing protein tyrosine kinase [Trifolium pratense]
MMGSEEREKVVKKKIQELEEGHAFMKQEMSKLKFNDGKEQQHRQRSQSVSPQRSRLGSWNKGLPSHINIY
PGHLTTDVRLLNKRYLATDVGLLNKRNRSAENLYGYAEEEVLGQDGIDLLVDSRDWGLAHDTVNCVMKGE
RWSGQFLVKNKKGDSFLAVTTNTPFYDDGSLVGIISVSSDSRPFLEMRAPFFGVKNAESDNGSMRRSSSV
ANKLGFDSQQPLQSAIASKFSNLASKMSNKVKSRIWTGENNVDREGVNGESYHSELGFSEHREDANSSGD
TTPRGDLPQSPPGVFYRVEEKFEGKTLRDSGDESEGKPVHKILPTKAEAWIQKKTLSWPWRTNDREESEA
RNVRAADPWMQNDQENESVNQKNLSSGLKHESQAGESNRHTNNEGSLSRSSSYNVILNNTSSASSCGSAG
SGGVNSKVDVDSDCLDYEILWEDLTIREQIGQGCCGTVYHALWYGSDVAVKVFSKQEYSDDLIQS**FRQEV**
SVMKRLRHPNILLFMGAVTSPQRLCIVTEFLPRGSLCRLLHRNTSKLDWRRRVHMALDIARGVNYLHHYN
PPIIHRDLKSSNLLVDKNWTVKVGDFGLSRLKHETYLTTKTGRGTPQWMAPEVLRNEPSDEKSDVYSFGV
ILWELATEKIPWDNLNAMQVIGAVGFMNQRPEIPKDVDPGWASLIEICWHSDPTCRPTFPELLERLKELQ
KQYVIQFQAARSAGGESTQRKES
>XP_022138774.1 3-phosphoinositide-dependent protein kinase B-like [Momordica charantia]
MGTPPAEELLRKIRELEEGQAHLKKEMSKLLLSGTPADSNSEHQIHHHHHHQRSQSISPQRSRFATARRR
GGASGGLDAAAAAWKKGSASFRHSSPLQRESRTRDPLNAGGGGGCSGPAAVNFTNKQYLNILQSMGQSVH
IFDLNCRIIYWNRAAETVYGYSAAEALGQDAVGLLVDPEDFAIANDVLRFVTAGENWTGQFPVKNKMGQK
FVVVANNSPFYDDGALVGIICVSSDSRPFQELKIPLAVGSKQQDGADSSLVRSRSTVSTKLGLDPQQPLQ
VAIASKISNLASKVSNKVKSKIRTGENSSDREGGSGDSYHSDHGFPDAVLCDHREDANSSGASTPRGDST
AHGAFSQIEEKCSGKLVRDSGDEVKGKPTIQKILSSKAEEWIAKKGLSWPWKGTEQEGSEARTARFVWPW
VQNDQEAEPGNHKSFSSAGKLEMQQNDGHRAGNNEASGSWSSFNVNSTSSASSCGSTSSSAVNKVDMETD
CLDYEILWEDLTIGEQIGQGSCGTVYHALWYGSDVAVKVFSKQEYSDDVILS**FRQEVSLMKKLRHPNILL**
FMGVVTSPQRLCIVTEFLPRGSLFRLLQRNSGKLDWRRRTHMALDIARGMNYLHHCNPPIIHRDLKSSNL
LIDKNWTVKVGDFGLSRLKRDTYLTTKTGKGTPQWMAPEVLRNDPSDEKSDIYSFGVILWELATEKIPWE
NLNSMQVIGAVGFMNQRLEIPKDVDPQWASIIESCCNSEPANRPSFQVLIDRLRELQRKYAIQLQAARSG
GDNNHPQKET
>XP_020976484.1 serine/threonine-protein kinase STY17 isoform X1 [Arachis ipaensis]
MGTSEAKEEELVKKIEKLEAGHVQLKHEMSKLKLSERRHHHRHRSHSLSPQRSRLGGCGGGGVAAAGCNN
KGSSSSPLKRESRSSNNHNHNHNQNQNQNENGDGDGDGDGDVGVNVGLSEKQYMNILQSMGHSLHILDLQ
CRIIYWNPSAENLYGYAAVEALGKDGIELLVDPIDFGLAYDVFNHVTVGESWSGQFPVKNKMGEKFSVVA
TNTPFYDDDRKLIGVISVSIDSRPFLETRATLSDVKNAAPGSDLDYNRAKSGISNKLGLDSQQPLQVALA
SKISNLASKVSNKVKSRMRTGENGVDRDGWSGESHHSDQSFSDCVFSDQKEDGNSSGASTPRGDVPPPFG
VFCHVEEKSPAKSSRDSGDDGEGKPIHKIITSKAEAWIQKRTLSWPWRGNGQAGSEARNVSVARPLVQND
QENELVNQMNLSSESRMEVQSGNRPVNNEASGSWSSVNVNSTSSTSSCGSGSSGAVNNKVDIDTDCLDYE
ILWEDLTIGEQIGQGSCGTVYHALWYGSDVAVKVFSKQEYSEDVILS**FRQEVSVLKRLRHPNIILFMGAV**
TSPQRLCIVTEFLPRGSLFRLLQRNTSKLDWRRRVHMALDIARGVNYLHRCSPPIVHRDLKSSNLLVDKN
WTVKVGDFGLSRLKHETYLTTKTGKGTPQWMAPEVLRNEPSDEKSDVYSFGVILWELATEKIPWDTLNAM
QVIGAVGFMNHQLEIPADVDPQWASIMESCWHRSNRRHGHPGLHRWVPPDPSPFPRHTPSLPSEAASSSL
PPLLPPDPHMGLLHHPPFLFFLLRPPPMLSVTANGINPRHR
>XP_015959519.1 serine/threonine-protein kinase STY46 isoform X2 [Arachis duranensis]
MGTSEAKEEELVKKIEKLEAGHVQLKHEMSKLKLSERRHHHRHRSHSLSPQRSRLGGCGGGGGAAAGCNN
KGSSSSPLKRESRNSNNHNHNHNQNQNQNEDGDGDCDGDGDGDVVGVNVGLSEKQYMNILQSMGHSLHIL
DLQCRIIYWNPSAENLYGYAAVEALGKDGIELLVDPIDFGLAYDVFNHVTVGESWSGQFPAKNKMGEKFS
VVATNTPFYDDDRKLIGVICVSIDSRPFLETRATLSDVKNAAPGSDLDYNHAKSGISNKLGLDSQQPLQV
ALASKISNLASKVSNKVKSRMRTGENGVDRYGWSGESHHSDQSFSDCVFSDQKEDGNSSGASTPRGDVPP
PFGVFCHVEEKSPAKSLRDSGDDGEGKPIHKIITSKAEAWIQKRTLSWPWRGNGQAGSEARNVPVARPLV
QNDQEHELVNQMNLSSESKMEVQAGNRPVNNEASGSWSSVNVNSTSSTSSCGSGSSGAVNNKVDIDTDCL
DYEILWEDLTIGEQIGQGSCGTVYHALWYGSDVAVKVFSKQEYSEDVILS**FRQEVSVLKRLRHPNIILFM**
GAVTSPQRLCIVTEFLPRGSLFRLLQRNTSKLDWRRRVHMALDIARGVNYLHRCSPPIVHRDLKSSNLLV
DKNWTVKVGDFGLSRLKHETYLTTKTGKGTIEISHWISDYAASRDFRSDVYSFGVILWELATEKIPWDTL
NAMQVIGAVGFMNHRLEIPADVDPQWASIMESCWHSDPAQRPTFQELVERLREMQRRHAIQLQAARSASG
GSNQKES

>XP_016197858.1 dual specificity protein kinase splA isoform X2 [Arachis ipaensis]
MGTSEAKEEELVKKIEKLEAGHVQLKHEMSKLKLSERRHHHRHRSHSLSPQRSRLGGCGGGGVAAAGCNN
KGSSSSPLKRESRSSNNHNHNHNQNQNQNENGDGDGDGDGDVGVNVGLSEKQYMNILQSMGHSLHILDLQ
CRIIYWNPSAENLYGYAAVEALGKDGIELLVDPIDFGLAYDVFNHVTVGESWSGQFPVKNKMGEKFSVVA
TNTPFYDDDRKLIGVISVSIDSRPFLETRATLSDVKNAAPGSDLDYNRAKSGISNKLGLDSQQPLQVALA
SKISNLASKVSNKVKSRMRTGENGVDRDGWSGESHHSDQSFSDCVFSDQKEDGNSSGASTPRGDVPPPFG
VFCHVEEKSPAKSSRDSGDDGEGKPIHKIITSKAEAWIQKRTLSWPWRGNGQAGSEARNVSVARPLVQND
QENELVNQMNLSSESRMEVQSGNRPVNNEASGSWSSVNVNSTSSTSSCGSGSSGAVNNKVDIDTDCLDYE
ILWEDLTIGEQIGQGSCGTVYHALWYGSDVAVKVFSKQEYSEDVILS**FRQEVSVLKRLRHPNIILFMGAV**
TSPQRLCIVTEFLPRGSLFRLLQRNTSKLDWRRRVHMALDIARGVNYLHRCSPPIVHRDLKSSNLLVDKN
WTVKVGDFGLSRLKHETYLTTKTGKGTPQWMAPEVLRNEPSDEKSDVYSFGVILWELATEKIPWDTLNAM
QVIGAVGFMNHQLEIPADVDPQWASIMESCWHSDPAQRPTFQELVERLREMQRRHAIQLQAARSASGGSN
QKES
>XP_020164533.1 dual specificity protein kinase shkD-like [Aegilops tauschii subsp. tauschii]
MDSSGEELLKKIRQLEVGQAQLKQEMSKFALPPTGGGGERRRSQSVSPSRGAPPHPAPAPAPSRRLSGGL
DGGQRGWGRGSASFSHSSPLQREGRAAAEGGATGAGLAERQYRRVLQSLGQSVHILDLDGRIIYWNRSAE
NLYGYPASEVLGQDALMLLVDSRDLSVVNDMFRRISLGESWTGKFPVKNKAGDRFLAVGTNTPFYDEDGS
LVGIICVSSDSRALEEILSGPSTSARSHSESSSPSCCDGSCSNSNRKTSLLNRNPFDPQQSTIASKITNL
ANKVTNKVRSRVKMDENGMVREGGSGESQCSDRDNKEGPSSGPSTPRGDAPHGAFGTEENSPGKSTKTNG
DESEAKIGLHKMLSSKAEALLNKKGITWPWKGRENDVPDCRNQVTLPSLHSEQENGQNHPKVSDTQLAEY
NQPNKNEASGSWSSFNNNSSSSASSTGSTNSSALYKIDHEADCLDYEILWEDLVIGEQVGQGSCGTVYHA
LWYGSDVGVKVFSRQEYSEEVIQA**FRQEVSLMKKLRHPNILLFMGAVTSPHRLCIVTEF**LPRGSLFRLLQ
RSTTKLDWRRRVHMALDVARGMNYLHHYSPPIIHRDLKSSNLLVDKNWTVKVADFGLSRLKRETYLTTKT
GKGTPQWMAPEVLRNEPSDEKSDVYSYGVILWELVTQKIPWENLNSMQVIGAVGFMNQRLEIPSETDPYW
TSLILSCWETDPQSRPSFQELLEKLRELQRKYAVQNQVQRNASAAAKNSIIEE
>XP_006280140.1 mitogen-activated protein kinase kinase kinase 13 isoform X4 [Capsella rubella]
MAERLYGFSAAEALGKDPIDILVDVQDASVAQNITRRCSSGESWTGEFPVKNKAGDRFSVVTTMSPSYDD
DGSLIGIICITNDSALFQDPRGPPAKTRGKEGETSFSRVTSSVASKLGLDSKEAVVSKLGLDSQQPIQVA
IASKISDLASKVGNKVKSKIRAGDNNSSHFEGGSGESHQSDQGFFDAAFCDRREDAATSGANTPRGDFIQ
SPFGVFLSSNEKASSKPFRDSSDENDGNSGTPKTLTSKAEEWMVKKGLSWPWKGNEREGLEGRRGHSVWP
WVQNEQQKEQAYQSNSNHSVKSESQACESIKASSNEPMGYWSSSVNVNSTSSTSSCGSTSSSVMNKVDID
TDCLDYEILWEDLTIGEQIGQGSCGTVYHGLWFGSDVAVKVFSKQEYSEEIITS**FRQEVSLMKRLRHPNV**
LLFMGAVTSPQRLCIVTEFLPRGSLFRLLQRNTSKLDWRRRIHMASDIARGMNYLHHCTPPIIHRDLKSS
NLLVDRNWTVKVADFGLSRIKHETYLTTKTGRGTPQWMAPEVLRNEAADEKSDVYSFGVILWELVTEKIP
WENLNAMQVIGAVGFMNQRLEVPKNVDPKWISLMESCWHSEPQHRPSFQEIMEKLRELQRKYTIQFQAAR
AASIDNSALKGK
